# Genome-wide analysis of the grapevine stilbene synthase multigenic family: genomic organization and expression profiles upon biotic and abiotic stresses

**DOI:** 10.1186/1471-2229-12-130

**Published:** 2012-08-03

**Authors:** Alessandro Vannozzi, Ian B Dry, Marianna Fasoli, Sara Zenoni, Margherita Lucchin

**Affiliations:** 1 Department of Agronomy, Food, Natural resources, Animals and Environment, University of Padova, Agripolis, viale dell’Università 16, 35020, Legnaro, Padova, Italy; 2 Centro Interdipartimentale per la Ricerca in Viticoltura ed Enologia, Università di Padova, Agripolis, viale dell’Università 16, 35020, Legnaro, Padova, Italy; 3 CSIRO Plant Industry, PO Box 350, Glen Osmond, SA, 5064, Australia; 4 Dipartimento di Biotecnologie, Università degli Studi di Verona, Strada Le Grazie 15, 37134, Verona, Italy

**Keywords:** Stilbene synthase, Chalcone synthase, Abiotic stress, Downy mildew, Grapevine

## Abstract

**Background:**

Plant stilbenes are a small group of phenylpropanoids, which have been detected in at least 72 unrelated plant species and accumulate in response to biotic and abiotic stresses such as infection, wounding, UV-C exposure and treatment with chemicals. Stilbenes are formed via the phenylalanine/polymalonate-route, the last step of which is catalyzed by the enzyme stilbene synthase (STS), a type III polyketide synthase (PKS). Stilbene synthases are closely related to chalcone synthases (CHS), the key enzymes of the flavonoid pathway, as illustrated by the fact that both enzymes share the same substrates. To date, STSs have been cloned from peanut, pine, sorghum and grapevine, the only stilbene-producing fruiting-plant for which the entire genome has been sequenced. Apart from sorghum, STS genes appear to exist as a family of closely related genes in these other plant species.

**Results:**

In this study a complete characterization of the STS multigenic family in grapevine has been performed, commencing with the identification, annotation and phylogenetic analysis of all members and integration of this information with a comprehensive set of gene expression analyses including healthy tissues at differential developmental stages and in leaves exposed to both biotic (downy mildew infection) and abiotic (wounding and UV-C exposure) stresses. At least thirty-three full length sequences encoding VvSTS genes were identified, which, based on predicted amino acid sequences, cluster in 3 principal groups designated A, B and C. The majority of VvSTS genes cluster in groups B and C and are located on chr16 whereas the few gene family members in group A are found on chr10. Microarray and mRNA-seq expression analyses revealed different patterns of transcript accumulation between the different groups of VvSTS family members and between *VvSTSs* and *VvCHSs*. Indeed, under certain conditions the transcriptional response of VvSTS and VvCHS genes appears to be diametrically opposed suggesting that flow of carbon between these two competing metabolic pathways is tightly regulated at the transcriptional level.

**Conclusions:**

This study represents an overview of the expression pattern of each member of the STS gene family in grapevine under both constitutive and stress-induced conditions. The results strongly indicate the existence of a transcriptional subfunctionalization amongst *VvSTSs* and provide the foundation for further functional investigations about the role and evolution of this large gene family. Moreover, it represents the first study to clearly show the differential regulation of VvCHS and VvSTS genes, suggesting the involvement of transcription factors (TFs) in both the activation and repression of these genes.

## Background

Approximately 450 million years ago, several pioneering green algal ancestors, probably related to Charales [1], spread out from water to occupy a new bio-geographical niche: dry land. This colonisation of dry land was accompanied by the need to deal with important stresses including desiccation, UV radiation, as well as attack by already diversified microbial soil communities. This led to a number of physiological adaptations, including the evolutionary emergence of entirely new specialized secondary metabolic pathways [2]. One in particular was crucial: the phenylpropanoid pathway, which represents a ubiquitous and specific trait of land plants providing vital compounds such as lignin and flavonoids [3]. Lignin is a structural polymer important for the structural integrity necessary for the emergence of self-supporting structures. Flavonoids, which often impart a species-specific chemical ‘signature’ upon an organism, serve vital roles in the protection of plants against biotic and abiotic stresses, reproduction and internal regulation of cell physiology and signalling [4].

The role of phenylpropanoid compounds in defence appears to be restricted to a minor class of compounds that are often referred to as phytoalexins. The term “phytoalexins” probably derives from the Greek language and means “warding off agents in plants” and refers to low mass, lipophilic, antimicrobial compounds that not only accumulate rapidly at the site of interaction with incompatible pathogens [5,6] but also accumulate in response to abiotic stresses such as exposure to UV light, wounding or treatment with chemicals such as salts and heavy metals, respiratory inhibitors and surfactants [7]. Because of the agricultural and economic importance of grapevine as a crop plant, the strategies it uses to defend against phyto-pathogenic organisms, as well as deal with abiotic stresses, has attracted considerable interest in recent times. Amongst the arsenal of defence mechanisms available to grapevine cells is the production of phytoalexins. Phytoalexins from the Vitaceae family have been the subject of numerous studies over the past decade, not only because of their biological activities *in planta,* but also because of their possible pharmacological applications.

Although phytoalexins display an enormous chemical diversity throughout the plant kingdom, in grapevine they constitute a rather restricted group of molecules belonging to the “stilbene family” [8]. Plant stilbenes, together with flavonoids, belong to the class of compounds called polyketides, which represents a major group of phenylpropanoids derived from the extension of the activated form of coumaric acid with three acetyl moieties. Apart from the Vitaceae, stilbenes have been detected in at least 72 unrelated plant species distributed among 31 genera and 12 families including Fagaceae, Liliaceae, Moraceae, Myrtaceae, Papilionaceae, Pinaceae, and Poaceae [8-10]. Despite the multiplicity of forms detected in these different plants, most plant stilbenes, including those ones detected in grapevine, are derivatives of the basic unit *trans*-resveratrol (3,5,4’-trihydroxy-*trans*-stilbene). In addition to resveratrol, more complex compounds derived from its modification have also been detected in grapevine such as *cis*- and *trans*- piceid [11-14], viniferins, which represent oligomers arising from the oxidative coupling of resveratrol, pterostilbene [15,16] and piceatannol [17].

Several plant species, such as *Polygonum cuspidatum* and *Pinus* spp. constitutively accumulate large amount of stilbenes [18-23]. However, the majority of studies conducted on cells and leaves of peanut, grapevine and pine seedlings have shown that stilbenes are present at only very low levels under normal conditions, but strongly accumulate in response to a wide range of biotic and abiotic stresses as a result of an increased transcription of their biosynthetic genes and the co-ordinated activation of upstream genes belonging to the general phenylpropanoid pathway, such as *PAL* and *C4H*. These abiotic stress treatments include mechanical damage [24,25], UV-C light irradiation [26,27], treatments with chemicals such as aluminium ions, cyclodextrins and ozone [28-30] and the application of plant hormones like ethylene and jasmonates [31-33]. In terms of biotic stresses, the biosynthesis of stilbenes in grapevine tissues is also particularly well documented, with the accumulation of stilbenic compounds reported following infection with a range of different pathogens, including powdery mildew (*Erysiphe necator*) [34,35], downy mildew (*Plasmopara viticola*) [36], gray mold (*Botrytis cinerea*) [16,37,38] and *Aspergillus carbonarius*[39,40].

**Figure 1:**
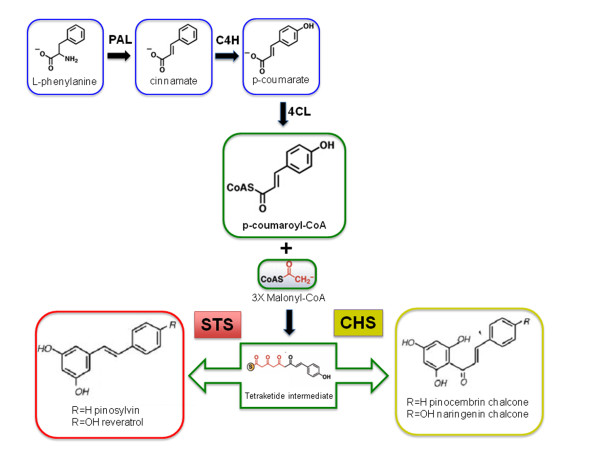
**General phenylpropanoid pathway and flavonoid and stilbene branching pathways.** The enzymes shown in these pathways are as follows: PAL, phenylalanine ammonia-lyase; C4H, cinnamate-4-hydroxylase; 4CL, 4-cumaroyl: CoA-lyase; CHS, chalcone synthase; STS, stilbene synthase.

Stilbene synthase (STS) is the key enzyme leading to the biosynthesis of resveratrol and stilbenes and was firstly extracted and purified from stressed cell suspension cultures of peanut (*Arachis hypogaea*) [41]. It belongs to the type III polyketide synthase super family, of which chalcone synthase (CHS) represents the archetypal enzyme. The enzyme is a dimer of estimated molecular weight 90 kDa with an iso-electric point (pI) of 4.8. A conserved cysteine residue, located in the central section of these proteins has been shown to be essential for the catalytic activity of both STS and CHS enzymes and represents the binding site for the p-coumaroyl-CoA starting substrate [42]. The region around this active site is well conserved and can be used as a signature pattern for CHS and STS. The two proteins show a high degree of similarity based on sequence homology (which reaches approximately 75-90% amino acid sequence identity depending on the species), and on the comparison of their crystallographic structures [43], suggesting that STS independently evolved from CHS several times in the course of evolution [44]. STSs, which, in contrast with the ubiquitous CHSs, are only present in stilbene-producing plants, catalyse the formation, in a single enzymatic reaction, of exactly the same linear tetraketide intermediate (from p-coumaroyl-CoA and three malonyl-CoA) produced by CHS in the flavonoid pathway, but with a different cyclization that leads to the production of stilbenes rather than chalcones (Figure 1).

To date, STS genes have been cloned from peanut (*A. hipogaea*), Scots pine (*P. sylvestris*), Eastern white pine (*P. strobus*), Japanese red pine (*P. densiflora*), grapevine (*V. vinifera* L.) and sorghum (*Sorghum bicolor*). In many of these plant species STSs exist as a family of closely related genes. For example, two STS genes have been found in peanut and Eastern white pine [45,46], Scots pine has a small multigene family of at least five pynosylvin synthase genes (*PST1PST2PST3PST4* and *PST5*) [47] and Japanese red pine possesses three members (*PdSTS1PdSTS2* and *PdSTS3*) [48]. Apart from Sorghum, for which only one STS member has been identified [10,49], grapevine represents the only stilbene producing plant species for which the entire genome has been sequenced [50,51]. Forty-three VvSTS members were predicted with GAZE and JIGSAW prediction tools in the 8.4 X coverage genome draft of the PN40024 genotype (French-Italian consortium) [50] while only twenty-one members were predicted from the genome sequence of the PN ENTAV 115 genotype (IASMA) [51]. Sparvoli et al. [52] performing a molecular characterization of structural genes involved in anthocyanins and stilbene biosynthesis in *V. vinifera* has previously hypothesized that these gene families probably arose from the same ancestral gene and that subsequent gene duplications and molecular divergence may have contributed to the establishment of functionally distinct genes.

This aim of this study was to clarify the genome organization of the entire STS family in grapevine and investigate the transcriptional response of each VvSTS member in different grapevine tissues, at different developmental stages and under different stress conditions, in order to determine if this gene family evolved into different sub-groups characterized by specific role in the response to different stresses or in the plant development.

## Results

### Identification, annotation and chromosomal distribution of grapevine STS genes

The genome sequence of the near-homozygous PN40024 genotype of the *V. vinifera* cv. Pinot noir was searched for predicted STS gene sequences. These were predicted on the genome draft by combining *ab initio* models together with *V. vinifera* complementary DNA sequences, such as EST databases and alignment of gene/protein models from other species [50]. The Hidden Markov Model (HMM) for the CHS/STS active site [PS00441] was obtained from PROSITE and used in a BLASTP search against the 8.4X, 12X V0 and 12X V1 proteome databases. In order to extend the search to identify putative gene family members not predicted by the GAZE and JIGSAW software programs, a tBLASTx search of the HMM and of the entire amino acid sequence of previously identified VvSTS was also performed against the genome sequence. Fifty-one hits were obtained. Three predictions carrying the CHS/STS HMM were found to encode for chalcone synthase genes and were excluded leaving a total of forty-eight putative VvSTS gene sequences. These sequences were designated as *VvSTS1* to *VvSTS48* based on their chromosomal position (Table 1). *VvSTS1-6* are located in a region of approximately 90 Kb on chr10, whereas *VvSTS7-48* reside on chr16, within a 500 Kb region. Five sequences corresponding to genes designated as *VvSTS11VvSTS14VvSTS34VvSTS40* and *VvSTS44* fall in genome regions which are not predicted to contain any gene based on GAZE and JIGSAW prediction tools.

**Table 1:** **Grapevine*STS*members identified based on the PN40024 12X V1 coverage**

**Proposed nomenclature**	**Chr**	**PN40024 12X V1 Location**	**Closest prediction 12X V1**	**ORF predicted**
*VvSTS1*	10	14216112..14217677	Vv10s0042g00840	Unsure
			Vv10s0042g00850	
			Vv10s0042g00860	
*VvSTS2*	10	14246945..14248453	Vv10s0042g00870	No
*VvSTS3*	10	14264038..14265601	Vv10s0042g00880	Unsure
			Vv10s0042g00890	
*VvSTS4*	10	14284187..14285750	Vv10s0042g00910	Unsure
*VvSTS5*	10	14298957..14300520	Vv10s0042g00920	Yes
*VvSTS6*	10	14304787..14306350	Vv10s0042g00930	Yes
*VvSTS7*	16	16239028..16240564	Vv16s0100g00750	Yes
*VvSTS8*	16	16252494..16254029	Vv16s0100g00760	Unsure
*VvSTS9*	16	16268816..16270352	Vv16s0100g00770	Yes
*VvSTS10*	16	16276570..16278105	Vv16s0100g00780	Yes
*VvSTS11*	16	16285039..16284807	-	No
*VvSTS12*	16	16287922..16286924	Vv16s0100g00800	Unsure
*VvSTS13*	16	16290882..16289536	Vv16s0100g00810	Unsure
*VvSTS14*	16	16324386..16323781	-	No
*VvSTS15*	16	16335697..16337233	Vv16s0100g00830	Yes
*VvSTS16*	16	16344516..16343202	Vv16s0100g00840	Yes
*VvSTS17*	16	16347949..16346580	Vv16s0100g00850	Yes
*VvSTS18*	16	16351428..16350059	Vv16s0100g00860	Yes
*VvSTS19*	16	16368410..16366907	Vv16s0100g00880	Yes
*VvSTS20*	16	16387529..16386013	Vv16s0100g00900	Yes
*VvSTS21*	16	16398234..16399770	Vv16s0100g00910	Yes
*VvSTS22*	16	16406519..16405205	Vv16s0100g00920	Yes
*VvSTS23*	16	16409837..16408469	Vv16s0100g00930	Yes
*VvSTS24*	16	16413317..16411948	Vv16s0100g00940	Yes
*VvSTS25*	16	16431392..16430833	Vv16s0100g00950	Unsure
*VvSTS26*	16	16440652..16441618	Vv16s0100g00960	No
*VvSTS27*	16	16468549..16467015	Vv16s0100g00990	Yes
*VvSTS28*	16	16478613..16477097	Vv16s0100g01000	Yes
*VvSTS29*	16	16493131..16491597	Vv16s0100g01010	Yes
*VvSTS30*	16	16505168..16503636	Vv16s0100g01020	Yes
*VvSTS31*	16	16509479..16507942	Vv16s0100g01030	Yes
*VvSTS32*	16	16511216..16512602	Vv16s0100g01040	Yes
*VvSTS33*	16	16521936..16520374	Vv16s0100g01060	No
*VvSTS34*	16	16523861..16523409	-	No
*VvSTS35*	16	16527862..16526326	Vv16s0100g01070	Yes
*VvSTS36*	16	16557435..16555945	Vv16s0100g01100	Yes
*VvSTS37*	16	16588984..16587447	Vv16s0100g01110	Yes
*VvSTS38*	16	16608730..16607176	Vv16s0100g01110	Yes
*VvSTS39*	16	16617258..16615702	Vv16s0100g01120	Yes
*VvSTS40*	16	16620545..16618991	-	No
*VvSTS41*	16	16624624..16623088	Vv16s0100g01130	Yes
*VvSTS42*	16	16629091..16627536	Vv16s0100g01140	Yes
*VvSTS43*	16	16645747..16644190	Vv16s0100g01150	Yes
*VvSTS44*	16	16649027..16647473		No
*VvSTS45*	16	16675524..16673986	Vv16s0100g01160	Yes
*VvSTS46*	16	16684264..16682709	Vv16s0100g01170	Yes
*VvSTS47*	16	16699842..16698303	Vv16s0100g01190	Yes
*VvSTS48*	16	16711818..16710281	Vv16s0100g01200	Yes

Genes have been named from *VvSTS1* to *VvSTS48* based on the chromosomal location. The chromosomal location and corresponding identifier on the 12X V1 coverage are also provided. The predicted open reading frame (ORF) prediction was assigned as follows: Yes: genes encoding a complete ORF; Unsure: genes for which it was not possible to determine with certainty whether they encode a full length or truncated ORF based on available sequence information from the PN40024 and PN ENTAV 115 genotypes and specifically matching paired-end reads obtained from the whole-transcriptome sequencing of Pinot noir clone 115; No: genes encoding for a truncated ORF.

Although the Genoscope integrated method for deducing proteins is very exhaustive, some gene models were found to be incorrect based on available EST sequences and when compared with cloned VvSTS CDS sequences already deposited on the GenBank database. With particular reference to the 12X V1 coverage assembly, the predictions designated as Vv10s0042g00840, Vv10s0042g00850 and Vv10s0042g00860 are listed as three different genes in the proteome database, but our analysis indicates they represent one single unique VvSTS gene, designated as *VvSTS1* (Table 1). A similar observation was made for the predictions Vv10s0042g00880 and Vv10s0042g00890, which also represent a single gene designated as *VvSTS3*. The opposite situation was observed for genes designated as *VvSTS37* and *VvSTS38*, which are represented by the same prediction Vv16s100g01110. Genomic sequences of five genes, *VvSTS12*, *VvSTS13*, *VvSTS14*, *VvSTS25* and *VvSTS26* were obtained by corresponding sequences from the PN ENTAV 115 genome sequence because of gaps in the PN40024 assembly.

Based on the deduced amino acid sequences obtained from Genoscope predictions and from manual analysis using Vector NTI software, several VvSTS proteins were found to be truncated because of SNP/mutations leading to premature stop codons or in/del mutations causing frame-shifts and changes in the protein primary structure. In order to investigate whether these observations were limited to the PN40024 genotype used by the French-Italian Consortium or were also detectable in other genotypes, the closest sequences in the PN ENTAV 115 genotype and specifically matching paired-end reads obtained from the whole-transcriptome sequencing of Pinot noir clone 115, were screened for these mutations (Additional file 1). Based on available sequence information it was not possible to determine with absolute certainty whether *VvSTS1*, *VvSTS3*, *VvSTS4*, *VvSTS8*, *VvSTS12, VvSTS13* or *VvSTS25* encode a complete ORF. *VvSTS2*, *VvSTS33*, *VvSTS40* and *VvSTS44* were predicted to have premature stop codons in all three genotypes screened or in both the PN40024 and PN ENTAV 115 where no specific paired-end reads were available. *VvSTS11* and *VvSTS34* represent gene fragments of 233 nt and 453 nt respectively with the upstream and downstream sequences not coding for STS. Finally *VvSTS18* was predicted to be a coding gene based on the fact that at least one allele at this locus was predicted to encode a complete ORF based on the three genotypes sequences screened.

The genomic sequences of the VvSTS genes detected in the PN40024 genome ranged in size from a minimum length of 1315 nt (*VvSTS16*) to a maximum of 1566 nt (*VvSTS1*) depending on the length of the single introns present in all members within the triplet coding for Cys-60. Deduced protein length for all 36 full-length coding genes was 392 aa (Additional file 2), whilst of those pseudogenes that possessed the CHS/STS active site (R-[LIVMFYS]-x-[LIVM]-x-[QHG]-x-G-C-[FYNA]-[GAPV]-G-[GAC]-[STAVK]-x-[LIVMF]-[RAL]) *VvSTS1* encodes for a 234 aa product, *VvSTS2* for a 206 aa product (181 without considering the first 46 nt which are probably wrongly predicted), *VvSTS4* for a 267 aa product and *VvSTS18* for a 185 aa product. All other genes give products lacking the active site and were considered non functional.

### Phylogenetic analyses of the deduced VvSTS proteins

**Figure 2:**
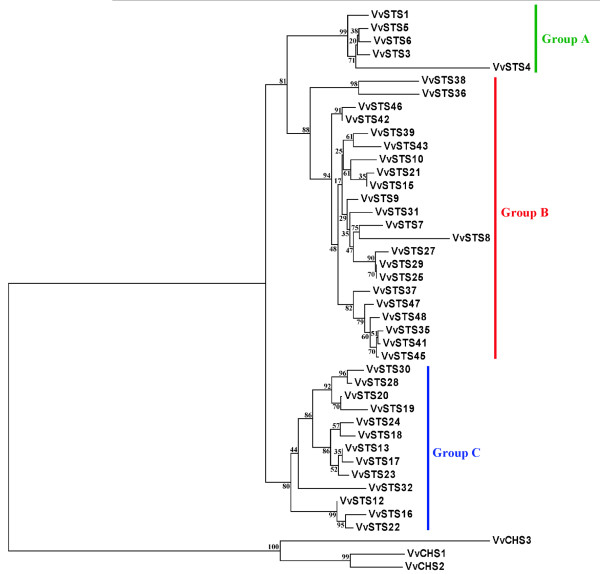
**Phylogenetic tree of predicted*STS*proteins in grapevine.** Consensus phylogenetic tree generated after sequence alignment with MAFFT 6.0 using the neighbour-joining method. VvSTS gene members predicted to encode for a truncated ORF were not considered. Deduced protein for *VvCHS1*, *VvCHS2* and *VvCHS3* were also included in the analysis. Reliability of the predicted tree was tested using bootstrapping with 1000 replicates. Numbers at the forks indicate how often the group to the right appeared among bootstrap replicates. Different coloured bars indicate three main sub-groups designated as A, B and C.

In order to examine the phylogenetic relationship between the predicted VvSTS proteins a phylogenetic tree was constructed using the E-INSI tool of the MAFFT 6.0 software as described in the tutorial for the Grapevine Genome Annotation (http://www.vitaceae.org/index.php/Annotation_tutorial) provided by the International grape Genome Program (IGGP) steering committee. Gene members encoding a truncated ORF were not included in the alignment, but for VvSTS genes where both coding and non-coding alleles had been identified in different genotypes, the coding ORFs were also included in the analyses. The three VvCHS proteins corresponding to VvCHS1 [Genbank: AB015872], VvCHS2 [Genbank: AB066275] and VvCHS3 [Genbank: AB066274], respectively Vv14s0068g00930, Vv14s006800920 and Vv05s0136g00260 in the 12X V1 assembly of the PN40024 genotype, were also included in the analysis to ascertain the evolutionary relationships between VvSTS and VvCHS proteins. Figure 2 shows that VvSTS proteins cluster in three main sub-families, which have been designated as groups A, B and C. Group A is composed entirely of those members located on the chr10 (i.e. *VvSTS1-6*), while groups B and C are composed of 22 and 13 members respectively all positioned on chr16. The three VvCHS proteins were found to cluster outside the tree as outgroups.

### Microarray analysis of VvSTS and VvCHS expression during grapevine development and post-harvest berry withering

**Figure 3:**
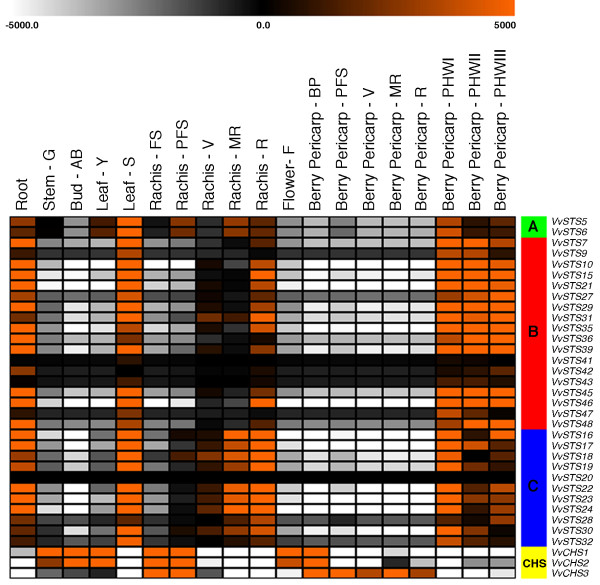
**Expression image of the complete*VvSTS*family in the*V. vinifera*cv Corvina atlas.** Expression data was normalised based on the mean expression value of each gene in all tissues/organs analysed. Different organs/tissues are displayed vertically above each column. VvSTS gene names are displayed to the right of each row and are clustered in different groups A, B, C according to protein homology as shown in Figure 2. Expression data for VvCHS genes are included for comparison. The colour scheme used to represent expression level is orange/white: black boxes indicate a low variation in expression, white boxes indicate a decrease and orange boxes indicate a increase respect to the mean value of a given gene. Y, young leaf; FS, fruit-set; S, senescence; G, green stem; AB, bud-burst; PFS, post fruit-set; V, véraison; MR, mid-ripe; R, ripe; F, flowering (50% cap-fall); PHWI, post-harvest withering I (1^st^ month); PHWII, post-harvest withering II (2^nd^ month); PHWIII, post-harvest withering III (3^rd^ month).

The expression pattern of VvSTS genes encoding a complete ORF were analysed in a global *V. vinifera* cv. Corvina gene expression atlas of different organs at various developmental stages (Fasoli *et al.*, in preparation). Although *VvSTS37* and *VvSTS38* were predicted to encode a full-length protein, these genes were excluded from the expression analyses as they are represented by the same prediction in the reference genome, which could lead to incorrect estimations of the expression values for these genes. The expression atlas was generated using a microarray technology based on gene predictions obtained from the 12X V1 coverage assembly of the PN40024 genotype. Figure 3 shows a graphical representation of the expression pattern of each VvSTS, together with the three VvCHS genes identified in grapevine, and was generated using MeV software. Raw VvSTS and VvCHS expression values are reported in Additional file 3.

The first thing to note regarding the results shown in Figure 3 is that the majority of VvSTS gene family members show little or no constitutive expression in most grapevine tissues including young leaves, stems, buds, flowers and developing grape berries. The exceptions to this appear to be roots and all stages of rachis development in which members of all three VvSTS groups show elevated levels of constitutive expression. As a group, members of VvSTS group A also appear to have a higher level of constitutive expression in young leaf (Y) tissues than the majority of members of subgroups B and C.

Another important observation regarding VvSTS expression in developing grape tissues is that members of all groups are strongly induced during aging or senescence. This was observed in both senescing leaves and in berries undergoing the process of berry withering. Berry withering is a post-harvest drying process used specifically with Corvina berries for the production of dessert and fortified wines. The drying process leads to alterations in most quality characteristics and an increase in the concentration of simple sugars. Berries were sampled for expression analysis after the first, second and third month of the withering phase. The results clearly show a very strong induction of nearly all VvSTS family members in the berry pericarp in response to the withering process. This organ appears to accumulate VvSTS transcripts within the exocarp tissue, whereas the expression is much lower in berry flesh (Additional file 4).

Figure 3 also shows a comparison between the constitutive expression patterns of the VvSTS family members with the three VvCHS genes in grapevine. What is clear from this comparison is that the expression of the three VvCHS genes appears to show an opposite pattern to that of the VvSTS genes across a number of different tissues and developmental stages. For example, expression of at least one member of the VvCHS gene family is found to be high in young leaves, stems, buds, the rachis at fruit set and in developing berries in which VvSTS expression is generally very low. The converse is also true: in tissues where VvSTS expression is strongly induced e.g. senescing leaves, *in vitro* roots, the rachis from ripe berries and withering berries, there is little or no VvCHS expression.

### mRNA-seq analysis of VvSTS and VvCHS expression in grape leaves in response to stress

The same VvSTS and VvCHS gene sequences predicted in the PN40024 genome sequence and analysed in the grapevine expression atlas (Figure 3) were also studied for their expression under biotic and abiotic stress conditions. In order to overcome the difficulty posed by the high sequence conservation between these genes, which makes it difficult to clearly discriminate between individual members using PCR-based expression analyses, a whole transcriptome (mRNA-seq) approach was performed using the Illumina Next Generation Sequencing (NGS) technology. *V. vinifera* cv Pinot noir leaf discs were collected at 0, 24 and 48 h after wounding, UV-C exposure and infection with *P. viticola*. Seven pools of RNA samples, representing each treatment and the control sample, which was common for all three treatments, were used to build libraries for high-throughput parallel sequencing using an Illumina Genome Analyser II (GAIIx). Each treatment was represented at least by 32 million reads, a tag density sufficient for quantitative analysis of gene expression [53].

**Figure 4:**
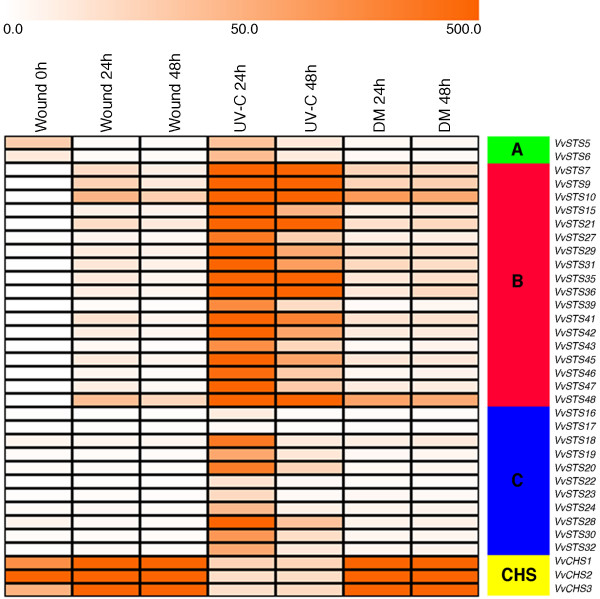
**Expression image of the complete*VvSTS*gene family in*V. vinifera*cv. Pinot noir stressed leaves.** The treatments (wounding, exposure to UV-C and downy mildew infection) are displayed vertically above each column. Genes are displayed to the right of each row and clustered in different groups A, B, C and CHS as evidenced by different colours. Relative levels of expression are indicated by a colour gradient from low (white) to high (orange). Expression data are expressed as the number of mapped reads per Kb of exon per million mapped reads (RPKM).

All three stress treatments resulted in a significant induction of expression of at least some members of the VvSTS gene family (Figure 4, Additional file 5). Of the three stress treatments employed, UV-C exposure led to the highest induction of the majority of VvSTS members, followed by downy mildew infection and wounding. The wounding and UV-C responses appeared to peak within 24 h of treatment whereas the downy mildew-treated discs continued to show an increase in VvSTS transcription after 48 h, presumably reflecting the establishment of the downy pathogen within the leaf tissue. Interestingly, there appeared to be only minor differences in VvSTS transcription between wounded and downy mildew-inoculated discs after 24 hours indicating that VvSTS genes are not induced in the early stages of downy mildew infection prior to haustorial formation.

In agreement with the microarray data shown in Figure 3, VvSTS genes in group A, unlike those in groups B and C, are characterised by significant levels of constitutive expression in young leaves. Furthermore, group A genes are not induced in response to wounding and show only a minor increase in transcription in response to UV-C treatment compared to control discs. In contrast, VvSTS genes in group B are highly responsive to abiotic stress treatments with wounding resulting in increases in transcription ranging from 7 to 186 fold after 24 h. When these discs are also exposed to UV-C light there is a further increase in transcription ranging from 11.3 to 27 fold. VvSTS genes in group C appear to show transcriptional responses which are intermediate between those of genes in groups A and B.

The relationship between VvSTS and VvCHS transcription in young leaf tissues subjected to abiotic stress treatments (Figure 4) appears to be somewhat more complicated than was observed for constitutive expression patterns in different grapevine tissues (Figure 3). As observed with VvSTS genes in groups B and C, wounding led to an increase in transcription of all three VvCHS genes ranging from 3.2-8.7 fold after 24 h. However, in accordance with the inverse transcriptional responses of VvSTS and VvCHS genes observed in a range of different grapevine tissues in Figure 3, the further increase in VvSTS transcription in response to UV-C treatment was accompanied by an 8–20 fold reduction in expression of the VvCHS genes (Figure 4) to levels below that found in control discs.

### Quantitative RT-PCR analyses of selected VvSTS members of groups A, B and C *and VvCHSs* in stressed leaves

**Figure 5:**
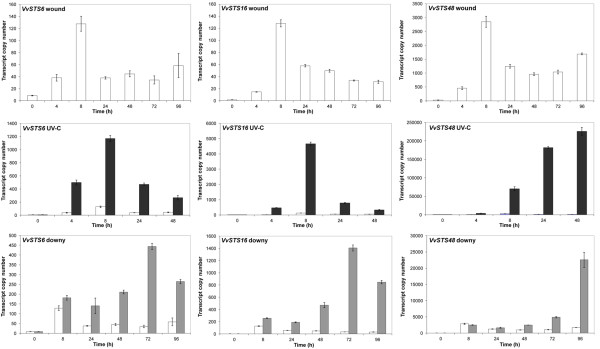
**Expression of grapevine selected*VvSTS6*,*VvSTS16*and*VvSTS48*genes upon abiotic and biotic stresses.** Selected members representative for A- (*VvSTS6*), B- (*VvSTS48*) and C- (*VvSTS16*) subgroups were screened by quantitative RT-PCR in wounded, UV-C exposed and downy mildew infected samples. Transcript are normalised to the expression of elongation factor (*EF1*) and plotted as actual transcript copy number. Bars indicate standard error (SE) in three technical replicates. Empty bars represent wounded samples, which also represent the control for the other treatments. Dark gray bars represent UV-C treated samples. Light gray bars represent downy mildew infected samples.

**Figure 6:**
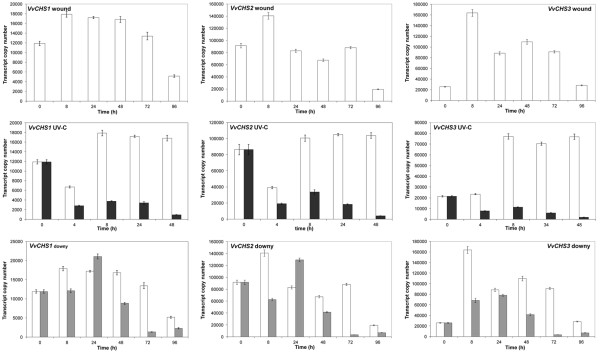
**Expression of grapevine*CHS*genes upon abiotic and biotic stresses.** Grapevine *CHS1*, *CHS2* and *CHS3* genes were screened by quantitative RT-PCR in wounded, UV-C exposed and downy mildew infected samples. Transcript are normalised to the expression of elongation factor (*EF1*) and plotted as actual transcript copy number. Bars indicate standard error (SE) in three technical replicates. Empty bars represent wounded samples, which also represent the control for the other treatments. Dark gray bars represent UV-C treated samples. Light gray bars represent downy mildew infected samples.

The analysis of mRNA-seq data from leaf samples treated by wounding, UV-C exposure and *P. viticola* infection, together with analysis of gene expression atlas in *V. vinifera* cv. Corvina indicated there are differential expression patterns across different VvSTS groups and between members of the VvSTS and VvCHS polyketide synthase families. To confirm and investigate these observations in more detail, the expression patterns of selected members of the VvSTS groups A, B and C i.e. *VvSTS6*, *VvSTS48* and *VvSTS16* and the three grapevine VvCHS genes was monitored using quantitative RT-PCR across a time course series following wounding, UV-C irradiation, and *P. viticola* inoculation of Shiraz leaf tissue (Figures 5 &6).

**Figure 7:**
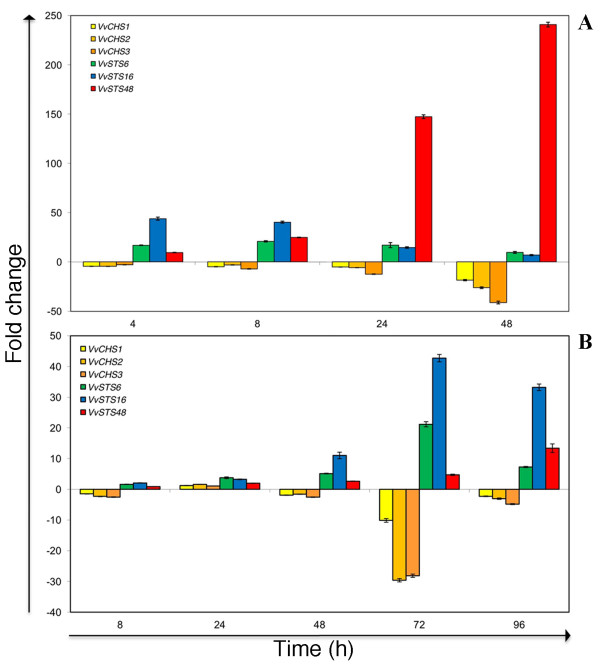
**Relative changes in expression of*VvSTS*and*VvCHS*genes in response to UV-C irradiation and downy mildew infection.** This figure summarizes the fold changes of selected *VvSTS6*, *VvSTS16* and *VvSTS48* and *VvCHSs* genes in UV-C exposed (A) and downy mildew infected leaf discs (B). Fold change was obtained by calculating the ratio between treated (UV-C or downy infected) and untreated (i.e. wounded discs) samples at the same time point.

Elongation factor *EF1* was selected as the reference gene as it was found to be more stable than 18 S and actin in the wounded and UV-C irradiation treatments (data not shown). Figure 5 illustrates changes in selected *VvSTSs* mRNA transcript levels over time in response to the three applied stresses. The qPCR results confirmed the results of the mRNA-seq experiment with the group B gene *VvSTS48* showing much higher levels of transcript accumulation than VvSTS genes in groups A (*VvSTS6)* and C and (*VvSTS16)* respectively under all stress treatments. However, it is clear from this more detailed expression analysis that *VvSTS6* and *VvSTS16* display a similar pattern of induction in response to these three stress treatments and that the pattern of induction of these group A and C genes is clearly different to that observed for the group B gene, *VvSTS48*. For example, both *VvSTS6* and *VvSTS16* show a peak of transcription at 8 h post UV-C treatment. In contrast, *VvSTS48* shows a continual increase in transcript levels over the whole 48-h period following UV-C treatment. This is well illustrated in Figure 7A, which shows a comparison of the fold-change in gene expression in response to UV-C treatment for these three VvSTS genes. Based on this analysis it would appear that *VvSTS6* and *VvSTS16* actually respond earlier than *VvSTS48* to UV-C treatment. However, it should be noted that the transcriptional activity of *VvSTS48* is such that the level of transcription of this gene in response to wounding alone, at 4 h, is still greater than that observed for *VvSTS6* and *VvSTST16* at 4 h following wounding plus UV-C treatment (Figure 5). Indeed, 8 h after UV-C treatment the level of expression of this group B gene is approximately 60 and 15 fold higher than is observed for the subgroup A and C VvSTS genes respectively.

Differences in the timing of response of these three VvSTS group representatives are also evident from the downy mildew inoculation experiment. In agreement with the mRNA-seq data (Figure 4) there appears little transcriptional response from three VvSTS genes to downy mildew infection within the first 24 h. However, from 48 hpi both *VvSTS6* and *VvSTS16* show a marked increase in transcription peaking at 72 hpi (Figures 5 &7B). In contrast, a significant increase in downy-mildew induced transcription is not observed for *VvSTS48* until 72 hpi and continues to increase up to 96 hpi. Even so, the copy number of this group B gene transcript at 72 hpi is still significantly higher than that observed for the group A and C genes (Figure 5).

Figure 6 shows the patterns of expression observed for the three *VvCHSs* genes in response to the same treatments. As observed in both the Corvina gene expression atlas (Figure 3) and the Pinot noir mRNA-seq analysis (Figure 4), constitutive levels of expression of all three VvCHS genes are much higher than VvSTS genes in young Shiraz leaves (cf. Figures 5 &6). Both *VvCHS1* and *VvCHS2* show only a minor increase in expression upon wounding, although a slight decrease in expression was detected at 96 h after treatment. In contrast, *VvCHS3* showed a 5-fold increase at 8 h after wound treatment, followed by a decrease in expression as observed for the other two VvCHS genes.

Of greater significance is the observation that the application of both the UV-C and downy treatments led to a significant reduction in transcript accumulation of all three VvCHS genes compared to control (wounded-only) discs (Figure 6). This is most clearly displayed in Figure 7, which show that as the combined level of VvSTS transcription increased in response to these biotic and abiotic stress treatments, so the level of transcription of all three VvCHS genes was suppressed by as much as 18–41 fold at 48 h post UV-C treatment (Figure 7A) and 10–30 fold within 72 h of downy mildew inoculation (Figure 7B).

## Discussion

### Expansion of the STS family in grapevine

To date, STS genes have been cloned from several plant species including peanut, sorghum, pine and grapevine [54]. In peanut and pine STS genes are organised in multigenic families composed of 2–5 members, although in the absence of a whole genome sequence for these species an accurate estimate of the number of family members is difficult. Grapevine and sorghum represent the only two species which possess stilbene biosynthetic genes for which the genomes have been completely sequenced. Screening of the sorghum genome sequence revealed the presence of a single, unique STS gene [10; 49]. In this study, a search for STS genes in the most update version of the genome assembly of the grape PN40024 genotype referred to as 12X V1, led to the identification of 48 members, designated *VvSTS1* to *VvSTS48* and included at least 33 full-length coding genes, 8 pseudogenes and 7 sequences that remain to be resolved (Table 1).

The striking size of the grapevine STS gene family, compared to other stilbene-producing plant species, is not surprising given that analysis of the grape genome sequence has already indicated an expansion in the size of other gene families related to secondary metabolism in grapevine [50,51]. For example, it is estimated that there may be up to 35 terpene synthase (*TPS*) genes in grapevine based on the genome assembly of the PN ENTAV 115 genotype [51]. The phenylalanine ammonia-lyase (*PAL*) gene, which encodes for the key enzyme of the phenylpropanoid pathway, has 13 members in grapevine, whereas only 4–8 genes are present in Arabidopsis, rice, and poplar [51]. More recently, Falginella et al. [55] reported on the expansion and subfunctionalization of the grapevine flavonoid 3’,5’-hydroxylase (*F3’5’H*) gene family, responsible for the biosynthesis of precursors of blue anthocyanins. Large-scale (segmental or whole) genome duplication has been recurring during angiosperm evolution and is one of the driving forces in the evolution of genomes and genetic systems [56,57]. Subsequent gene loss and gene rearrangements further affect gene copy number and fractionate ancestral gene lineages across multiple chromosomes. The expansion of the *F3’5’H* family, which is composed of 16 members, appears to be the result of multiple events of segmental and tandem duplications that occurred in the Vitaceae lineage, after the separation from other dicots [55]. Of the 16 copies of *F3'5'H*s present in the PN40024 genome, 15 reside in a tandem array within a 650 Kb region on chr 6 with an isolated copy on chr 8. Although a detailed study of the VvSTS evolution was not the major aim of this study, the model proposed for the *F3’5’H* family could also be applied to the VvSTS gene family. The majority of VvSTS members (*VvSTS7VvSTS48*) are located in a 500 Kb region on chr16, which shows numerous paralogous zones, not only at the level of coding regions, but also in non-coding regions (data not shown) suggesting multiple events of tandem and segmental duplication. Something similar could have happened for members *VvSTS1VvSTS6* located within an 80 Kb region of chr 10. A recent analysis of the genome architecture of the PN40024 line and its high-identity duplication content by [58], identified that 85 Mb out of the 487 Mb comprising the grapevine genome is duplicated. Furthermore, they found that chr 16, which contains the majority of VvSTS family members, has the highest percentage (25.08%) of segmental duplication among the assembled non-random chromosomes.

It is noteworthy that duplicate genes involved in secondary metabolism or involved in the response to exogenous stimuli, appear to be more frequently maintained than duplicate genes belonging to other categories [59-61]. Moreover it’s generally assumed that the maintenance of duplicate genes provides a foundation for consolidation and refinement of established functions, particularly in secondary metabolism, by preserving extra copies that guarantee a gene reservoir for adaptive evolution [62-64]. What is particularly interesting in the case of the STS gene family is that the majority of plants don’t even possess a single STS gene, whilst grapevine has evolved such a large STS gene-reservoir. The fact that a single STS gene is present in the monocot Sorghum [10,49] suggests that the evolution of STS from *CHS*, the common ancestor of PKSs, occurred before the monocot-dicot separation. Nevertheless, it’s difficult to explain the lack of stilbene-producing genes in the majority of plant species and the conservation and retention of many duplicated STS genes in a restricted group of unrelated species. It could be argued that the production of stilbenes did not confer an evolutionary advantage in those species that lost their biosynthetic genes or, on the other hand, that the majority of species were not able to cope with the production of compounds such as resveratrol, that, although related to benefits at low range of concentrations, are phytotoxic to plant cells at higher concentrations [65].

### Structure/Function of VvSTS proteins

All full-length VvSTS coding genes were found to encode proteins of 392 amino acids in length and contain the conserved CHS/STS active site (Additional file 2). In some cases (Table 1), it was not possible, based on currently available sequence information, to determine with certainty whether the genes encode for a complete or truncated ORF. This includes the genes *VvSTS1* and *VvSTS4*, which are particularly interesting as they possess the conserved CHS/STS active site within the truncated allele (Additional file 6).

In a previous study, which compared the enzymological properties of three STS proteins (PdSTS1, PdSTS2 and PdSTS3) and one CHS protein (PdCHSX) from Japanese red pine, it was observed that PdSTS3, which has a frame-shift mutation leading to a premature stop codon, presents a functional divergence compared to the other full-length STS/CHS proteins [47]. In particular, the PdSTS3 protein showed poor solubility compared to PdSTS2, but despite being truncated, still demonstrated a high potential for pinosylvin production. Furthermore, neither pinosylvin nor pinocembrin inhibited the PdSTS3 activity *in vitro*, whereas these metabolites effectively inhibited the activity of both PdSTS2 and PdCHSX. Thus, although the truncated ORFs of *VvSTS1* and *VvSTS4* are shorter than that observed for PdSTS3 (Additional file 6) we cannot rule out the possibility that these truncated alleles may still contribute to stilbene synthesis biosynthesis in grape cells.

Together with the CHSs, STSs represent the most studied enzymes of the plant type III PKS proteins and for this reason this group is often referred as the CHS/STS type III PKS family. The two enzymes compete for the same substrates, share very close amino acid sequences, and possess very similar crystallographic structures [43]. Previous phylogenetic analyses of the STS and CHS families indicated that STSs of Scots pine, peanut and grapevine do not form a separate cluster, but instead cluster with the CHSs proteins from the same or related plants [44]. This observation, reinforced by the observation that only three amino acids exchanges were required within the N-terminal 107 aa of CHS to shift the activity to a STS-type function, suggests that STS may have evolved from *CHS* several times during the course of evolution [44]. In this study, the three *CHS* genes identified in the PN40024 genotype, based on clones previously isolated in Cabernet Sauvignon [66], were included in the phylogenetic analysis performed on the STS family to investigate whether any of the predicted VvSTS proteins cluster more closely to the VvCHS clade. Sequence alignment and phylogenetic tree analyses revealed the existence of 3 VvSTS clades or groups, designated as A, B and C (Figure 2). Group A is composed of genes located on chr10, whereas groups B and C are composed of members located on chr16. However, neighbour-joining analysis indicated that all predicted VvSTS proteins cluster separately from the three VvCHSs, suggesting a conservation of function amongst all VvSTS members. This observation is in agreement with a recent functional study in which 10 different VvSTS genes (including members of each group) were transiently expressed in tobacco and all led to an accumulation of resveratrol and stilbenes, with no evidence for the production of any other products (Parage et al, in preparation).

### Temporal and spatial patterns of STS gene expression in grapevine

Using an expression atlas of *V. vinifera* cv. Corvina (Fasoli *et al.*, in preparation), it was possible to investigate patterns of expression of all of the predicted coding members of the VvSTS and VvCHS gene families in different grapevine tissues and at different developmental stages (Figure 3).

Expression of the majority of VvSTS genes was found to be very low in most non-stressed grapevine tissues analysed. The two exceptions to this were *in vitro* roots and the berry rachis. The high level of VvSTS expression in *in vitro* roots is in agreement with the detection of high levels of oligostilbenes in this organ [67]. Moreover, the propagation of this organ *in vitro* is an artificial procedure that could represent a stress for the plant, leading to the stress-induced transcription of VvSTS genes as observed in Figure 4. The elevated levels of *VvSTSs* expression in the berry rachis, however, are more surprising. What is particularly striking is the dramatic increase in transcription of group B and C VvSTS genes in the rachis during maturation of the Corvina berries while there is no detectable induction of VvSTS genes in the berries themselves (Figure 3). As discussed in more detail below, VvSTS expression in grape tissues such as leaves and berries appears to be strongly associated with senescence. Thus, the results shown in Figure 3 may reflect the fact that the rachis on Corvina berries undergoes maturation and senescence during berry ripening. This is also supported by the fact that rachis are generally brown, dehydrated and lignified by the time berries reach full maturity.

Interestingly, the microarray results did not show any significant increase in VvSTS expression in Corvina berries during both véraison and ripening. This is in contrast with previously reported studies, which indicate that healthy grape berries synthesise stilbene compounds under natural environmental conditions [14,68-70]. However, stilbene production during berry ripening has been shown to be genotype dependent with “high” producers such as Pinot noir producing up to 20 ug resveratrol per g berry fresh wt at maturity [14] compared to a low producer like Corvina which was found to synthesize only 1.5 μg g^-1^ at harvest [68]. It would appear, therefore, that the microarray technique was not sufficiently sensitive to detect the low level changes in VvSTS expression during ripening of the Corvina berries.

In general, VvSTS expression was low in young grape leaves except for two VvSTS gene members of group A. However, as observed for the rachis, grapevine leaves also show a dramatic increase in VvSTS transcription as they reach maturity and begin to senesce (Figure 3). This was true of gene members of each VvSTS group with individual genes increasing by as much as 2 (*VvSTS5*-6) to 130 fold (*VvSTS9*) in senescing leaves compared to young leaves. Leaf senescence is an active and highly regulated process that involves an integrated response of leaf cells to age information and other internal and environmental signals [71]. It is accompanied by a decreased expression of genes related to photosynthesis and protein synthesis and an increase in the expression of hundreds of senescence-associated genes [71]. Many of these genes are associated with the remobilization of nutrients to other developing organs [72]. However, it is not immediately clear as to what role stilbene biosynthesis would play in such a process. The observation that a number of pathogenesis-related (PR) genes are induced during leaf senescence has lead to the suggestion that the senescence program might have incorporated features of the pathogen-defense response to protect the senescing leaf against opportunistic pathogens [73]. Alternatively, the induction of STS genes in senescing leaves may simply be a consequence of changes in the levels of various phytohormones including abscissic acid (ABA), salicylic acid (SA), jasmonates (JA) and ethylene which are known to play an important role in regulating leaf senescence and which have also been shown to be involved in the induction of stilbene biosynthesis. For example, treatment of Cabernet Sauvignon cuttings with Ethephon, an ethylene-releasing compound, resulted in an enhancement of both *PAL* and STS gene induction leading to an increase in phytoalexins biosynthesis by [31]. Similarly, JA, another key hormone in the senescence response, has been shown to induce high levels of STS transcription in cell cultures of *V. vinifera* cv. Cabernet Sauvignon [74]. Therefore, it is likely that the increased expression of STS genes during leaf senescence is related to an accumulation of hormones such as ethylene and jasmonates, which are well known to be involved in these particular plants developmental stages.

### Stress-induced VvSTS gene expression in grapevine tissues

The majority of previous studies on the accumulation of stilbene compounds and their biosynthetic genes performed on peanut and grapevine tissues, indicated that these genes are highly inducible in response to a number of biotic and abiotic stresses including mechanical damage [24,25], UV-C light irradiation [26,27], treatments with chemicals such as aluminium ions, cyclodextrins and ozone [28-30] and infection, including powdery mildew, downy mildew and gray mold [35-40]. Although these studies have made important contributions to our general understanding of the behaviour of stilbene biosynthetic genes, in light of the information we now have regarding the size of the VvSTS gene family and the strong sequence conservation amongst its members, the interpretation of some of this data needs to be reconsidered. To this end, we investigated the transcriptional response of all of the predicted coding members of the VvSTS and VvCHS gene families to three abiotic stress treatments (post-harvest drying, wounding and exposure to UV-C radiation) and one biotic treatment (downy mildew infection) using either grape berries or grape leaves.

The process of post-harvest berry drying (berry withering) involves harvesting of ripe grapes and allowing them to dry over a period of three months in a naturally ventilated room. Its primary purpose is to alter berry quality characteristics and increase the concentration of simple sugars in the production of dessert and fortified wines typical of the Valpolicella region in Italy. However, the drying of harvested grapes in this way results in a loss of over 30% of their weight through evaporation during this post-harvest treatment [30] and, as such, imposes a significant water stress on the berries. It also results in a dramatic induction of the majority of VvSTS genes (Figure 3) demonstrating that drying berries are still capable of undergoing a significant stress response. Versari et al. [68] previously observed an increase in the resveratrol content of skins sampled from Corvina berries which had undergone an artificial berry withering treatment. A later study by Zamboni et al. [30] showed that berry withering was associated with an increase in the transcription of a range of genes involved in hexose metabolism and transport, cell wall composition, and secondary metabolism including a number of VvSTS genes. Our data extends these original observations to show that nearly all of the VvSTS gene members are markedly induced by the dehydration stress. Furthermore the increase in VvSTS expression was detected predominately within skin of the drying grape berry (Additional files 3 and 4). This is in agreement with the immuno-detection of STS proteins performed on berry extracts by Fornara et al. [75] who showed that STS protein is located mainly in berry exocarp during the véraison phase and is detected only occasionally within the mesocarp.

In order to obtain more control over the stress treatments imposed, the second set of experiments employed young rapidly expanding leaves harvested from glasshouse-grown *V. vinifera* cv. Pinot noir plants and utilised whole transcriptome mRNA-seq analysis to investigate the expression patterns of all of the predicted coding members of the VvSTS and VvCHS gene families in response to mechanical wounding, UV-C exposure and downy mildew (*P. viticola*) infection. In agreement with data obtained from the Corvina expression atlas (Figure 3), there appears to be a much higher level of constitutive expression of the group A VvSTS gene family members (*VvSTS5* and *VvSTS6*) than VvSTS gene members belonging to groups B and C raising the question as to the role of group A VvSTS proteins in young leaves. In terms of stress-induced expression, the results indicate that among the three stress treatments examined, UV-C exposure resulted in the highest VvSTS induction, followed by downy mildew infection and wounding (Figure 4), confirming previous observations [76]. The much larger increase in VvSTS induction in response to UV-C exposure may reflect the much larger number of cells within the leaf disc that are subjected to UV-C exposure compared to the wounding and downy mildew treatments which are only affecting a subset of cells. The data also indicates that members within the same VvSTS groups are not only related through protein homology (Figure 2) but also appear to show similar transcriptional responses (Figure 4). Thus, members of group B showed the highest response to all stress treatments, whereas group C members showed a reduced response, while the two group A genes showed little or no transcriptional response to the three stress treatments imposed.

In an attempt to validate the different stress-induced transcriptional responses within the VvSTS gene family, a more detailed analysis of individual members of group A (*VvSTS6*), group B (*VvSTS48*) and group C (*VvSTS16*) was undertaken using qPCR (Figure 5). The qPCR analysis confirmed the significant differences in the quantitative response of these different group members to the different abiotic and biotic stress treatments observed using the mRNA-seq analysis (Figure 4). At the peak of induction, the transcript copy number of *VvSTS48* was found to be 15–50 fold higher than the levels of *VvSTS16* and *VvSTS6*. If one assumes there are no major differences in translational efficiency between these different transcripts, this means that the bulk of the observed increase in the biosynthetic capacity of the stilbene pathway under stress conditions would appear to be contributed by the group B VvSTS family members.

Not only did qPCR analysis of stress-induced VvSTS induction in grape leaves confirm the quantitative differences in the transcriptional response of the different group members, it also demonstrated clear differences in the pattern and timing of the response to the different abiotic and biotic stress treatments. The transcriptional response of *VvSTS6* and *VvSTS16* to both UV-C treatment and downy mildew infection appears to be similar and more rapid than the response of *VvSTS48* (Figures 5 &7) leading one to speculate that the genes within the VvSTS groups A and C may be responding to different transcriptional signals to those in group B. The differential timing in the stress-response of VvSTS genes from the different groups provides an explanation for previous observations that total STS transcription in grape cells, as detected with Northern blot assays or PCR using generic primers, following stress or elicitor treatment, is often observed to be biphasic [27,76,77]. Indeed, Wiese et al. [77] previously suggested that the biphasic nature of the VvSTS response indicated that the VvSTS gene family may be divided into two groups: some expressed early with rapid degradation of the mRNA and others which are expressed later, providing more stable mRNA.

The different patterns of transcriptional response between the VvSTS groups further suggest that these genes may be responding to different signalling pathways. Both the JA and ethylene signalling pathways have previously been shown to have a role in STS transcription [31-33,74,78,79]. Faurie et al. [80] were able to show that co-treatment of Cabernet sauvignon suspension cells with methyl-jasmonate (MeJ) + Ethephon (ethylene) not only led to both a higher level of total stilbenes and VvSTS transcription compared to treatment with either elicitor alone, but also resulted in a biphasic pattern of transcription which was not observed in cells treated with MeJ or Ethephon only. These observations lend support to the hypothesis that VvSTS genes within the different groups respond to different stress/defense signalling pathways.

Transcriptional subfunctionalization has also been reported between the 15 members of the *F3’5’H* family [55], where the development of structural variation in the promoter regions of recently duplicated gene copies has led to differences in member-specific patterns of accumulation across organs, developmental stages and cultivars. Indeed, in the absence of transcriptional subfunctionalization, it would be hard to explain the retention of so many functionally identical VvSTS gene family members.

One question yet to be resolved is the identity of the transcription factor(s) which regulate VvSTS transcription. The expression of phenylpropanoid pathway genes is regulated by the binding of R3R3-type MYB transcription factors (TFs) to highly conserved *cis*-elements in their promoters [81,82]. Over the last few years a number of R2R3-type MYB TFs have been identified which regulate flavonol pathway genes in grapevine [83-87], however, to date, no transcription factor responsible for the regulation of VvSTS transcription has been reported. We have undertaken a PTM (Pavlidis Template matching) analysis of the whole mRNA-seq dataset for all 26,346 genes annotated in the 12X V1 PN40024 assembly to identify TF genes that show co-expression with VvSTS under the different stress conditions applied. This has resulted in the identification of two R3R3-MYB candidates which we believe have a role in the transcriptional regulation of the stilbene biosynthetic pathway (Vannozzi *et al.*, in preparation).

### Differential regulation of VvSTS and VvCHS genes in grapevine during development and in response to stress

Although there appears to have been little divergence in sequence since the evolution of STS from CHS, there has been sufficient mutation to lead to changes in the products synthesised. These products clearly fulfill very different roles in plant growth and development. Chalcone synthase catalyses the first committed step of the flavonoid biosynthetic pathway, which leads to the synthesis of anthocyanins, tannins and flavonols. Stilbene synthase, on the other hand, appears to function primarily as a stress-response protein, and has been implicated to have a role in defence against pathogens including powdery mildew, downy mildew and *Botrytis cinerea*[88,89]. As these two proteins represent branch points in the same pathway, the diversion of carbon skeletons into either secondary metabolism via CHS or stilbenic defence compounds via STS would be expected to be under tight control.

Evidence for the existence of crosstalk between these two pathways in grapevine cells is clearly evident from the analysis of gene expression data in Corvina tissues at various developmental stages (Figure 3). Tissues in which VvSTS expression levels are generally low i.e. stem, bud, young leaves, rachis at fruit set and developing berries are characterised by high constitutive expression of at least one of the three different VvCHS genes (Figure 3). Conversely, expression of all three VvCHS genes is suppressed in tissues in which VvSTS transcription is strongly induced i.e. roots, senescing leaves, maturing rachi and berries undergoing withering treatment. A similar pattern of inverse expression patterns between the members of the VvSTS and VvCHS gene families is also evident in grape leaves exposed to UV-C or inoculated with downy mildew (Figure 7). While both stress treatments resulted in dramatic increase in VvSTS transcription, the expression of all three VvCHS genes was strongly suppressed relative to the untreated leaf discs.

While a number of previous studies have shown that the expression of *CHS* can be induced by UV-A and UV-B light and pathogen infection (reviewed in [90]), this is the first study, to our knowledge, that has investigated the effect of UV-C light on *CHS* transcription. The other major difference between our study and previous investigations is that our research has been carried out on grapevine which has a highly evolved stilbene biosynthetic pathway which is strongly induced by both UV-C and downy mildew infection. As such, one might expect there to be an enhanced level of cross-talk between the flavonoid and stilbene biosynthetic pathways in grapevine.

It has been well documented that the triggering of defence pathways in plants causes a suppression of genes associated with photosynthesis and basic metabolism leading to the suggestion that there is a diversion of metabolic resources from general metabolism to defense-related metabolism, during pathogen attack. This is particularly true for the flavonoid pathway, which has been shown to be suppressed in a number of different plant species following exposure to fungal pathogens or fungal elicitors [91-94]. Recently Schenke et al. [95] demonstrated that the induction of biosynthetic pathways, in Arabidopsis, responsible for the synthesis of lignin and the phytoalexin scopoletin, by the bacterial elicitor flg22, was associated with a strong suppression of flavonol biosynthesis genes including CHS. They concluded that as flavonols, lignin and scopoletin are all derived from phenylalanine, that under stress conditions, the plant appears to refocuses it’s metabolism on the production of scopoletin and lignin, at the expense of flavonol. We propose that a similar antagonistic relationship exists between flavonol biosynthesis and stilbene biosynthesis in grapevine and that during periods of abiotic or biotic stress, stilbene biosynthesis takes precedence over flavonol biosynthesis.

How might this antagonistic relationship be regulated? In Arabidopsis, it appears that the antagonistic relationship between the flavonol and stress/defense biosynthetic pathways involves the action of at least two opposing MYB TFs: MYB12 (positive regulator) and MYB4 (negative regulator), which compete for binding to MYB-recognition elements within the promoters of the flavonol biosynthetic pathway genes. We are currently investigating whether R2R3-MYB candidates in grapevine might also repress the transcription of the VvCHS genes during the induction of the stilbene biosynthesis pathway.

## Conclusions

The sequencing of the grapevine genome, together with the vertiginous development of next generation sequencing technologies constitute a powerful tool for gene search and studies concerning their evolution, expression and function. This study embodies a particularly significant example of the advantages provided by these new tools, providing a detailed description of the expression patterns of each VvSTS genes in an extremely conserved gene family such as the one here described. This is the first study to our knowledge that describes the behaviour of the VvSTS gene family focusing on each single member and taking into account the strong sequence conservation that characterizes it. Using this approach we have demonstrated transcriptional subfunctionalization amongst different members of the VvSTS gene family. Furthermore we provide evidence for the co-ordinated transcriptional regulation of the VvSTS and VvCHS gene families which may serve to regulate the flow of carbon via these two competing metabolic pathways.

## Methods

### Grapevine tissues

For mRNA-sequencing analysis leaves were obtained from field grown vines at the “Lucio Toniolo” experimental farm of the University of Padova (Legnaro, PD, Italy). *V. vinifera* cv. Pinot noir plants (clone 115 on K5BB rootstock) were obtained from a certified nursery (Vitis Rauscedo, Pordenone, Italy). For quantitative RT-PCR analyses leaves of *V. vinifera* cv Shiraz were obtained and samples from potted glasshouse vines at the Waite Campus (Adelaide, South Australia, latitude 34°56’ south, longitude 138°36’ east). Grapevines were propagated from dormant cuttings obtained from the Riverland Vine Improvement Committee (Monash, South Australia).

### Database search, gene structure determination and chromosomal locations of grapevine STS genes

Protein sequences encoded by STS genes in grapevine were identified using BLAST [96] at the Genoscope BLAST server [97] providing the 8.4X and 12X V0 assembly coverage of the PN40024 genotype [50], and at the National Centre for Biotechnology Information (NCBI) [98]. The search was extended by consulting an uploaded version of the PN40024 12X assembly coverage, designated as V1, kindly provided by Prof. Giorgio Valle (University of Padova, Italy) [99]. A BLASTP search of the proteome database of the Genoscope Genome Project was carried out using the HMM (Hidden Markov Model) for the CHS/STS active site (PS00441) obtained from Prosite [100]. An e-value of 1e-3 was set to avoid false positives. To further increase the extent of the database search results, a tBlastN search of the genome sequence using one of the deduced protein sequences obtained from the Genoscope protein database was also performed in an attempt to capture VvSTS members that might have been missed using the GAZE and JIGSAW predictions and not included in the grapevine proteome database. Sequences were edited and analysed using Vector NTI v9 (Invitrogen) and gene structure was deduced from Genoscope gene annotation or from manual annotation based on the genomic sequences provided by Genoscope and comparison with the corresponding EST and deduced protein sequences for paralogous VvSTS genes. The chromosomal location of VvSTS genes was deduced using the BLAT server and additional physical localization tools at the Genoscope Genome Project website. Fragmentary predictions in the 12X PN40024 genomic sequence due to mistakes in the V1 assembly were substituted by corresponding sequences obtained from the parallel IASMA sequencing project obtained from the PN ENTAV 115 genotype [51] available at the NCBI database server.

### Phylogeny reconstruction and bootstrap analysis

A multiple sequence alignment (MSA) of the VvSTS deduced proteins, was performed using the E-INSI tool of the MAFFT 6.0 software [101], which takes into account the possibility of large gaps in the alignments. Three CHS proteins corresponding to CHS1 (AB015872; Vv14s0068g00930), CHS2 (AB066275; Vv14s0068g00920) and CHS3 (AB066274; Vv05s0136g00260) [66] were also included in the analysis. An unrooted phylogenetic tree was generated with the neighbour-joining method [102] using MEGA 5.0 software [103]. The best protein substitution model was chosen using the ProtTest suite [104]. Reliability of tree obtained was tested using bootstrapping with 1000 replicates. Resulting trees were edited and modified using Treedyn software (http://www.treedyn.org).

### Analysis of a gene expression atlas of *V. vinifera* cv. Corvina development

The expression patterns of VvSTS genes predicted from the analysis of the grapevine genome releases was analysed in a global *V. vinifera* cv. Corvina (clone 48) gene expression atlas of different organs at various developmental stages. Microarray data were kindly provided from Prof. Mario Pezzotti (University of Verona, Italy) for the following tissues: *in vitro* roots, green stem, buds after budburst (rosette of leaf tips visible), young leaves (leaves collected from shoots with only 5 leaves), senescing leaves (leaves at the beginning of leaf-fall), berry rachis (from fruit-set to ripening), flowers (50% cap-fall) and berry pericarp (from fruit set to ripe). In addition, berries were also examined which had undergone post-harvesting withering for 1–3 months after harvest. VvSTS genes encoding for an incomplete ORF were excluded from the analysis. Genes not represented by a 12X V1 identifier were also excluded. Data were analysed and expressed graphically by mean of MeV (Multi Experiment Viewer) software [105].

### mRNA-seq samples preparation and sequencing

For mRNA-seq analysis, leaf discs (15 mm diameter) were punched from healthy leaves detached from *V. vinifera* cv. Pinot noir glasshouse-grown vines. Discs were randomly selected from the third/forth leaves collected from different vines, subjected to abiotic and biotic stresses as described below and incubated upside down on moist 3MM filter paper in large Petri dishes. Punching of discs was considered as a wounding treatment *per se*, and as a control for other treatments. The UV-C treatment was achieved by exposing the abaxial surface of the discs to 30 W UV-C light for 10 mins at a distance of 10 cm. Downy mildew (*Plasmopara viticola*) infection was carried out spraying a solution containing downy mildew sporangia at concentration of 10^5^ sporangia ml^-1^. Pinot noir leaf discs were sampled at 0, 24 and 48 h after each treatment and total RNA extracted using the “Spectrum Plant total RNA Kit (Sigma) according to manufacturer’s instructions. RNA samples obtained from different plants were pooled, and 1μg of total RNA was retrotranscribed using the SuperScript III First Strand Synthesis System for RT-PCR (Invitrogen) with the oligo (dT)^20^ primer according to manufacturer’s instructions. The first-strand cDNA was initially analysed for the presence of VvSTS transcripts by PCR using the degenerate oligonucleotides GGTGACTAAGTCCGANCAYATGAC and GACTTTGGCTGTCCCCAYTCYTT designed using CODEHOP (Consensus-Degenerate Hybrid Oligonucleotide Primers) software [106] to ensure that the desired induction had been obtained. . Subsequently, 5 μg of the same RNA pools were used for mRNA-seq library preparation and Illumina® sequencing at the Institute of Applied Genomic (IGA, Udine, Italy). Each library had an insert size of 200 bp, and 36 to 39 bp paired ends reads sequenced on an Illumina Genome Analyzer IIx (GAIIx).

### Alignment and analysis of Illumina reads against the *V. vinifera* genome

Paired end reads obtained by Illumina mRNA-seq sequencing were aligned using both the 8.4X and 12X V1 coverage assembly of the PN40024 genotype sequence. Alignment of reads against the 8.4X reference genome assembly was carried out using CLC Genomic Workbench software (http://www.clcbio.com) at the Institute of Applied Genomics (IGA, Udine, Italy). Sequence alignment against the 12X V1 coverage, was performed using ELAND, an un-gapped alignment software package, which is part of the Illumina pipeline version 1.32. In both the alignments a maximum of two mismatches per read was set and, for an accurate measurement of gene expression, both unique reads and reads that occur up to ten times were included, to avoid underestimating the number of genes with closely related paralogues such as VvSTS. VvSTS members wrongly predicted (*VvSTS1VvSTS3VvSTS33* and *VvSTS34*) or encoding for an incomplete ORF were excluded from the analysis. Genes not represented by a 12X V1 identifier were also excluded. Data were analysed and expressed graphically by mean of MeV (Multi Experiment Viewer) software (http://tm4.org/mev/; [105]).

### Differential gene expression analysis

The evaluation of gene expression was performed on the mRNA-seq data obtained from the 8.4X and the 12X V1 coverage respectively with CLC Genomic workbench and ERANGE 3.1 programs [107]. In both cases, the transcriptional activity of each gene was defined as the number of mapped reads per kilobase of exon per million mapped reads (RPKM):

(1) $$RPKM=\frac{total\;exon\;reads}{mapped\;reads(million)\times exon\;length(Kb)}$$

Both programs compute the normalized gene locus expression level by assigning reads to their site of origin and counting them. In the case of reads that match equally in multiple loci, they are distributed proportionally to the weight of expression level given by specific single-matching reads. This means that if there are 10 reads that match two different genes with equal exon length, the two reads will be distributed according to the number of unique matches for these two genes. The gene that has the highest number of unique matches will thus get a greater proportion of the 10 reads. If a read has more hits than specified with this maximum number of hits setting, it will be ignored. Expression values were graphically represented using Multi Experiment Viewer software (MeV; http://www.tm4.org/mev/; [105]).

### Validation of mRNA-seq data by quantitative real-time PCR expression analysis

Leaf discs (15 mm diameter) were punched from healthy leaves detached from glasshouse-grown *V. vinifera* cv. Shiraz vines. Discs were obtained from leaves belonging to different plants and showing similar age based on size and node positions in plants, treated with the same different biotic and abiotic stresses previously described and incubated upside down on 3MM moist filter paper in large Petri dishes at 22°C under 12 h light / 12 h dark conditions until harvest at which point discs were immediately frozen in liquid nitrogen and stored at −80°C until RNA extraction. Five discs were randomly chosen from different treatments, at 0, 8, 16, 24, 48, 72 and 96 h after wound treatment, 0, 4, 8, 24 and 48 h after UV-C treatment and 0, 8, 24, 48 an 48 h after downy inoculation, dried with absorbent paper and immediately frozen in liquid nitrogen until extraction.

Selective primers were designed across dissimilar exonic DNA stretches or using a 3’-terminal SNP between the perfect match of the target gene-copy and the mismatched annealing site of paralogous sequences. Melt curve analysis, agarose gel electrophoresis, and DNA sequencing validated the absence of illegitimate cross-amplification of other paralogues. Expression analyses were carried by quantitative real-time PCR using a Sybr green method on a Rotor-Gene 3000 (Corbett Research, Mortlake, Australia) thermal cycler. Each 15ul PCR reaction contained 330 nM of each primer, 2ul of diluted cDNA, 1X FastStart Sybr green (Roche) and sterile water. The thermal cycling conditions used were 94°C for 10 min followed by 40 cycles of: 95°C for 30 s, 60°C for 30 s, and 72°C for 30 s, followed by a melt cycle with 1°C increments from 55 to 96°C. Real time PCR data processing was performed using the standard curve method. Standard curves were constructed using 10-fold serial dilutions, using cDNA from samples and stages in which the specific gene-copy was expressed or, if not possible, genomic DNA. In order to compare the expression level of different members belonging to the same PKS family, the actual transcript copy number was calculated based on the length of the product of amplification and its concentration in standard dilutions used to calculate the expression level. After testing the suitability of 18 S, actin and elongation factor EF1 for use of reference genes, elongation factor was selected for normalization of all samples analysed. The expression of each target gene was calculated relative to the expression of elongation factor in each cDNA using Rotor-Gene 6 Software (Corbett Research, Mortlake, Australia) to calculate CT values, observe melt profiles, extrapolate the concentration and measure primer pairs efficiencies. The primers used were: *VvSTS6*, VvSTS6F2 5’-GTTGTGCTGCATAGCGTTGC-3’ and 5’-GATTTAATTGGAAATTGTCCCCTTC-3’; *VvSTS16*, VvSTS16F2 5’-CTTTTGACCCAATTGGAATCAAC-3’ and VvSTS16R3 5’-TGACATGTTCCCATATTCACTTAG-3’; *VvSTS48*, VvSTS48F 5’-CTTGAAGGGGGAAAATGCT-3’ and VvSTS48R 5’-TTACTGCATTGAAGGGTA AACC-3’.

## Competing interest

Authors declare that in the past five years have not received reimbursements, fees, funding, or salary from an organization that may in any way gain or lose financially from the publication of this manuscript, either now or in the future. Authors do not hold any stocks or shares in an organization that may in any way gain or lose financially from the publication of this manuscript, either now or in the future. Authors do not hold or are currently applying for any patents relating to the content of the manuscript. Authors did not receive reimbursements, fees, funding, or salary from an organization that holds or has applied for patents relating to the content of the manuscript. Authors do not have any other financial or non-financial (political, personal, religious, ideological, academic, intellectual, commercial or any other) competing interest to declare in relation to this manuscript.

## Authors’ contributions

AV conceived the design of this study, planned and conducted most of the lab experiments, performed the bioinformatic data analysis and wrote the manuscript; IBD strongly contributed to the experimental planning, to the interpretation of results and participated in drafting the manuscript; MF and SZ analyzed and kindly provided data obtained from *V. vinfera* cv. Corvina expression Atlas; ML devised and supervised the study, contributed in interpretation of results and critically revised the manuscript. All authors have read and approved the final manuscript. The mRNA-seq data were submitted to Gene Expression Omnibus (NCBI) and are accessible through GEO accession number GSE37743.

## Supplementary Material

Additional file 1 **Description of mutations/SNPs in predicted*VvSTS*gene sequences from comparison of published grape genome sequences (PN40024 & PN ENTAV 115) and reads obtained from mRNA-seq analysis in this study.** Click here for file

Additional file 2 **Alignment of*VvSTS*and*VvCHS*protein sequences.** This figure shows the alignment of three entire VvSTSs deduced protein sequences representative of A- (VvSTS6), B- (VvSTS48) and C- (VvSTS16) groups with the three VvCHS proteins. The alignment was determined using MAFFT software and edited with GeneDoc software. The conserved CHS/STS active site is highlighted in green and differences in amino acid residues between VvSTS and VvCHS are highlighted in red. Click here for file

Additional file 3 **Robust Multichip Average (RMA) normalised expression data for selected*VvSTS*and*VvCHS*genes in the*V. vinifera*cv Corvina atlas.** Each hybridization was carried out on a NibleGen microarray 090818 Vitis exp HX12 (Roche, NimbleGen Inv., Madison, WI), representing 29549 predicted genes on the basis of the 12X grapevine V1 gene prediction version (http://srs.ebi.ac.uk/). The chip probe design is available at the following URL: http.//ddlab.sci.univr.it/FunctionalGenomics/. Normalised expression data here reported are limited to a subset of selected genes (VvSTS and VvCHS) and tissues from the whole data set (Fasoli *et al*., in preparation) and represent the averaged intensity of each gene in three biological replicates of each sample. A Pearson Correlation was previously carried out to evaluate the consistency of the biological replicates in each sample (R software). Y, young leaf; FS, fruit-set; S, senescence; G, green stem; AB, bud-burst; PFS, post fruit-set; V, véraison; MR, mid-ripe; R, ripe; F, flowering (50% cap-fall); PHWI, post-harvest withering I (1^st^ month); PHWII, post-harvest withering II (2^nd^ month); PHWIII, post-harvest withering III (3^rd^ month). Click here for file

Additional file 4 **Expression image of the complete*VvSTS*family in Corvina Berries undergoing withering process.** This picture illustrate more in detail the expression of VvSTS and VvCHS genes in berry tissues (skin and flesh) during the last developmental phases and withering process. Expression values are normalised based on the mean expression value of each gene in all tissues/organs analysed. Different organs/tissues are displayed vertically above each column. VvSTS gene names are displayed to the right of each row and are clustered in different groups A, B, C according to protein homology as shown in Figure 2. Click here for file

Additional file 5 ***VvSTS*and*VvCHS*genes RPKM expression data obtained from Illumina Genome Analyser II (GAII).** Here we report the RPKM expression values of all VvSTS and VvCHS considered in this study (Figure 4). Data shown were obtained by aligning paired end reads on the 12X V1 coverage assembly of the PN40024 genome sequence with ELAND, an ungapped alignment software package, which is part of the Illumina pipeline version 1.32. Differential gene expression analyses were performed by ERANGE 3.1 program. A maximum of two mismatches was set and, for an accurate measurement of gene expression, both unique reads and reads that occur up to ten times were included, to avoid underestimating the number of genes with closely related paralogues such as *VvSTSs*. Click here for file

Additional file 6 **Alignment of truncated*STS*protein sequences.** VvSTS1 and VvSTS4 deduced truncated proteins were aligned with a grapevine full-length STS (VvSTS48) and the three STS genes from *P.densiflora* (PdSTS1, PdSTS2 and PdSTS3). Alignment was obtained using MAFFT software and edited with GeneDoc software. The CHS/STS active site is highlighted in green. Stop codons are highlighted in red for those sequences considered of interest because they still contain the active site. Click here for file
