# Supplementary material for: Genome-wide analysis of the grapevine stilbene synthase multigenic family: genomic organization and expression profiles upon biotic and abiotic stresses
Source: BMC Plant Biol. 2012 Aug 3;12:130. doi: 10.1186/1471-2229-12-130 (PMC3433347; doi:10.1186/1471-2229-12-130)
Supplement: Additional file 1 — Description of mutations/SNPs in predictedVvSTSgene sequences from comparison of published grape genome sequences (PN40024 & PN ENTAV 115) and reads obtained from mRNA-seq analysis in this study. [file 1471-2229-12-130-S1.doc]

**Additional File 1**

Description of mutations/SNPs in predicted *VvSTS* gene sequences from comparison of published grape genome sequences (PN40024 & PN ENTAV 115) and reads obtained from mRNAseq analysis in this study.

***VvSTS*1**

This single gene is represented by 3 different predictions in the PN40024 12X V1 coverage: Vv10s0042g00840, Vv10s0042g00850 and Vv10s0042g00860.

PN40024: a “T” residue at nt position 700 in the predicted cDNA (nt 1087 in the predicted gene) determines a premature STOP codon at aa position 234;

PN ENTAV 115: the closest sequence to *VvSTS1* shows the same mutation causing a premature STOP codon at aa position 234;

PN mRNAseq: 48 specific reads matching with the PN40024 reference transcript in the region carrying the ambiguous nt; “T” residue is predicted in 42 reads (truncated ORF), “C” residue is predicted in 6 reads (complete ORF);

Prediction: *VvST1* ORF is unsure based on available sequence information as there is evidence from mRNA-seq data that there may be a full length allele expressed.

***VvSTS2***

PN40024: The first 46 nucleotides of predicted ORF do not code for a *VvSTS* protein. A single nucleotide deletion at position 477 of predicted cDNA (nt 868 of predicted gene starting from the first ATG upstream the STS coding sequence) determines a frame-shift mutation and a STOP codon at aa position 206 of deduced protein;

PN ENTAV 115: the first 46 nt of the closest sequence to *VvSTS2* encode for a correct STS. Single nucleotide (G) insertion at nt position 477 of cDNA (nt 869 starting from the STS ATG). Single nucleotide (C) insertion at position 498 of cDNA (nt 890 of the gene) determines a frame-shift mutation and a STOP codon at aa position 181 of deduced protein;

PN mRNAseq: no specific reads matching with PN40024 reference genome in the region carrying the deletion;

Prediction: *VvST2* is not coding for a complete STS ORF based on sequence information.

***VvSTS3***

This single gene is represented by two different predictions in the PN40024 12X V1 coverage: Vv10s0042g00880 and Vv10s0042g00890.

PN40024: “A” residue at nt position 458 of predicted cDNA (nt 843 in the predicted gene) determines a premature STOP codon at aa position 153 of deduced protein;

PN ENTAV 115: the closest sequence to *VvSTS3* shows a C residue at nt position 458 of predicted cDNA predicting a Serine residue at position 153 instead the stop codon and a complete ORF;

PN mRNAseq: 40 specific reads matching with PN40024 reference genome in the region carrying the SNP/mutation. A predicted in all 40 reads.

Prediction: *VvSTS3* gene is not predictable based on sequence information as there is evidence from PN ENTAV genotype there may be a full coding allele.

***VvSTS4***

PN40024: “A” residue at position nt 1101 of predicted cDNA (nt 1486 of predicted gene) determines premature STOP codon at aa position 367 of deduced protein;

PN ENTAV 115: the closest sequence to VvSTS4 carries a “R” [A or G] residue at the position nt 1101 of predicted cDNA encoding for both truncated and complete ORFs;

PN mRNAseq: no specific reads matching with PN40024 reference genome in the region carrying the SNP/mutation;

Prediction: *VvST4* ORF is unsure based on available sequence information as there is evidence from PN ENTAV 115 genotype there may be a full coding allele.

***VvSTS8***

PN40024: T at position nt 283 of predicted cDNA (nt 641 of predicted gene) causes a premature STOP codon at aa position 95;

PN ENTAV 115: C at the position nt 283 of predicted cDNA of the closest sequence to *VvSTS8* predicts a complete ORF;

PN mRNAseq: 101 specific reads matching with PN40024 reference genome in the region carrying the SNP/mutation. G predicted in 3 reads, T predicted in 98 reads;

Prediction: *VvSTS8* gene is unsure based on available sequence information as there is evidence from PN ENTAV 115 genotype there may be a full coding allele.

***VvSTS11***

PN40024: gene fragment. No prediction corresponding to this sequence on the 12X V1 coverage. The sequence is 233 nt long and correspond to the 3’end of the gene in the PN20024 coverage. The gene codifies for the last 76aa at the C-term of the protein;

PN ENTAV 115: the closest sequence to *VvSTS34* was confirmed to be a gene fragment;

PN mRNAseq: no specific reads matching with this gene fragment in the PN40024 reference genome;

Prediction: *VvSTS11* gene is not coding for a complete STS ORF.

***VvSTS12***

PN40024: sequence incomplete because of gaps in the assembly;

PN ENTAV 115: complete sequence encoding for a full length ORF;

PN mRNAseq: no specific reads matching with PN ENTAV VvSTS12 gene region;

Prediction: *VvSTS12* is not predictable based on available sequence information as there is only one sequence available.

***VvSTS13***

PN40024: sequence incomplete because of gaps in the assembly;

PN ENTAV 115: complete sequence encoding for a full length ORF;

PN mRNAseq: no specific reads matching with PN ENTAV VvSTS13 gene region;

Prediction: *VvSTS13* is not predictable based on available sequence information as there is only one sequence available.

***VvSTS14***

PN40024: sequence incomplete because of gaps in the assembly;

PN ENTAV 115: An insertion at position 84 of the deduced cDNA causing a frame-shift mutation and a premature STOP codon at aa position 35;

PN mRNAseq: no specific reads matching with PN40024 reference genome in the region carrying the insertion;

Prediction: *VvSTS14* gene is not coding for a complete STS ORF.

***VvSTS18***

PN40024: T at nt position 553 in the predicted cDNA (nt 744 in the gene) causes a premature STOP codon at aa position 185;

PN ENTAV 115: C at at nt position 553 in the predicted cDNA of the closest sequence to *VvSTS18* encodes for complete ORF;

PN mRNAseq: 73 specific reads matching with PN40024 reference genome in the region carrying the SNP/mutation. A predicted in 1 reads, C predicted in 20 reads, T predicted in 52 reads;

Prediction: *VvSTS18* gene is coding for a complete STS ORF as one genotype plus mRNAseq data support the presence of a full length ORF

***VvSTS25***

PN40024: sequence incomplete because of gaps in the assembly;

PN ENTAV 115: complete sequence encoding for a full length ORF;

PN mRNAseq: no specific reads matching with PN ENTAV VvSTS25 gene region;

***VvSTS26***

PN40024: the sequence is incomplete because of gaps in the assembly;

PN ENTAV 115: the closest sequence to VvSTS26 present a 5 nt insertion at position 101 of predicted cDNA causing a frame-shift and a premature STOP codon at aa position 62;

PN mRNAseq: no specific reads matching with PN ENTAV 115 reference genome in the region carrying the insertion

Prediction: *VvST26* is not coding for a complete ORF based on available sequence information as there is only one genome available where it encodes a truncated protein.

***VvSTS33***

PN40024: A single nucleotide deletion (C) at position 246 of the deduced cDNA and insertion (A) at position 741 cause a frame-shift that cover the protein for 109 aa.

PN ENTAV 115: The closest sequence to *VvSTS33* showed the same single nucleotide deletion (C) at position 246 of the deduced cDNA and insertion (A) at position 741.

PN mRNAseq: no specific reads matching with PN40024 reference genome in the region carrying the insertion;

Prediction: *VvSTS33* ORF is not coding for a complete STS ORF as it shows the same mutation in both genomes.

***VvSTS34***

PN40024: gene fragment. The sequence is 453 nt in length and correspond to the 3’end of the gene in the and codify for the last 151 aa at the C-term of the protein;

PN ENTAV 115: the closest sequence to VvSTS34 was confirmed to be a gene fragment;

PN mRNAseq: no specific reads matching with this gene fragment in the PN40024 reference genome;

Prediction: *VvSTS34* ORF is not coding for a complete STS ORF as it shows the same mutation in both genomes.

***VvSTS40***

PN40024: T at nt position 283 of the predicted cDNA causes a premature STOP codon at aa position 95. A single T at nt position 514 causes a second STOP codon at position 172;

PN ENTAV 115: T at nt position 283 of the predicted cDNA causes a premature STOP codon at aa position 95. A single T at nt position 514 causes a second STOP codon at position 172;

PN mRNAseq: no specific reads matching with this gene fragment in the PN40024 reference genome;

Prediction: *VvSTS40* ORF is not coding for a complete STS ORF as it shows the same mutation in both genomes.

***VvSTS44***

PN40024: T at nt position 283 of the predicted cDNA causes a premature STOP codon at aa position 95. A single T at nt position 514 causes a second STOP codon at position 172;

PN ENTAV 115: T at nt position 283 of the predicted cDNA causes a premature STOP codon at aa position 95. A single T at nt position 514 causes a second STOP codon at position 172;

PN mRNAseq: no specific reads matching with this gene fragment in the PN40024 reference genome;

Prediction: *VvSTS40* ORF is not coding for a complete STS ORF as it shows the same mutation in both genomes.
