# Supplementary material for: Genome-wide analysis of the grapevine stilbene synthase multigenic family: genomic organization and expression profiles upon biotic and abiotic stresses
Source: BMC Plant Biol. 2012 Aug 3;12:130. doi: 10.1186/1471-2229-12-130 (PMC3433347; doi:10.1186/1471-2229-12-130)
Supplement: Additional file 2 — Alignment ofVvSTSandVvCHSprotein sequences. This figure shows the alignment of three entire VvSTSs deduced protein sequences representative of A- (VvSTS6), B- (VvSTS48) and C- (VvSTS16) groups with the three VvCHS proteins. The alignment was determined using MAFFT software and edited with GeneDoc software. The conserved CHS/STS active site is highlighted in green and differences in amino acid residues between VvSTS and VvCHS are highlighted in red. [file 1471-2229-12-130-S2.pdf]

```
                   *        20         *        40         *        60         *        80
VvSTS6  : MASVEDIRNAQRAKGPATILAIGTATPDNCVYQSDYADYYFRVTKSEHMTDLKKKFNRICEKSMIKKRYIHLTEEMLEEH :  80
VvSTS16 : .....E............V...........L....F..............E.........D........S.......... :  80
VvSTS48 : .....EF.....................H.....................E.........D................... :  80
VvCHS1  : .V..AE..K....E....V........A.....A..P.....I.N.....E..E..K.M......N...M.....I.K.N :  80
VvCHS2  : .V..GE..KS...E....V........A.....A..P.....I.N.....E..E..K.M......N...M.....I.K.N :  80
VvCHS3  : .VT.NEV......E....VM.......P...D..T.P.....I.N...K.E..E..K.M.D........M.....I.K.N :  80

                   *       100         *       120         *       140         *       160
VvSTS6  : PNMGAYMAPSLNIRQEIITAEVPKLGKEAALKALKEWGQPKSKITHLVFCTTSGVEMPGVDYKLANLLGLETSVRRVMLY : 160
VvSTS16 : ..I........................................................A.................... : 160
VvSTS48 : ..I....................R..RD...............................A.................... : 160
VvCHS1  : ..VC.......DA..DMVVV..........V..I.....................D...A..Q.TK....KP..K.L.M. : 160
VvCHS2  : ..VC.......DA..DMVVV..........V..I.....................D...A..Q.TK....KP..K.L.M. : 160
VvCHS3  : ..VCE...A..DA..DMVVV..........A..I.....................D...A..Q.TK....RP..K.F.M. : 160

                   *       180         *       200         *       220         *       240
VvSTS6  : HQGCYAGGTVLRTAKDLAENNAGARVLVVCSEITVVTFRGPSETHLDSLVGQALFGDGSAAVIVGSDPDTSIERPLFQLV : 240
VvSTS16 : ............................................A...............T........L.......... : 240
VvSTS48 : ...........................................DA..............S.........V.......... : 240
VvCHS1  : Q...F.......L..........S..........A.......D...............A....I.A....K......E.. : 240
VvCHS2  : Q...F.......L.....................A.......D...............A..I.I.A....K......E.. : 240
VvCHS3  : Q...F.......L........K............A.......D...............A.........IPGV.K.M.E.. : 240

                   *       260         *       280         *       300         *       320
VvSTS6  : SAAQTFIPNTQGAIAGNLREVGLTFHLWPNVPTLISENIEKCLTQAFDPLGISDWNSLFWIAHPGGPAILDAIEAKLNLE : 320
VvSTS16 : ...........................................N.....I..N.................N.V....S.D : 320
VvSTS48 : .........SA.............................................................V....... : 320
VvCHS1  : .....IL.DSE...D.H..........LKD..G...K....S.VE..T.I.....................QV.L..G.K : 320
VvCHS2  : .....IL.DSE...D.H..........LKD..G...K....S.VE..K.I.....................QV.L..G.K : 320
VvCHS3  : .....IL.DSD...D.H..........LKD..G...K....S.NE..Q....K....I.............QV.E..A.K : 320

                   *       340         *       360         *       380         *
VvSTS6  : KKKLEATRHILSEYGNMSSACVLFILDEMRKKSLKEERTTTGEGLDWGVLFGFGPGLTIETVVLHSVVGATN- : 392
VvSTS16 : .Q..K....V...............M..........QK............................IPRDS.- : 392
VvSTS48 : .........V.........................G.NA...........................IPTV..- : 392
VvCHS1  : EE..R....V.......................IE.GKAS.....E............V........SAPPAH : 393
VvCHS2  : EE..R....V.......................IE.GKG......E............V.......LATQSTH : 393
VvCHS3  : PE..RS...V....................R..AE.GLK......E............V........ST---- : 389
```
