# Supplementary figures and images for: Genome-wide analysis of the grapevine stilbene synthase multigenic family: genomic organization and expression profiles upon biotic and abiotic stresses
Source: BMC Plant Biol. 2012 Aug 3;12:130. doi: 10.1186/1471-2229-12-130 (PMC3433347; doi:10.1186/1471-2229-12-130)

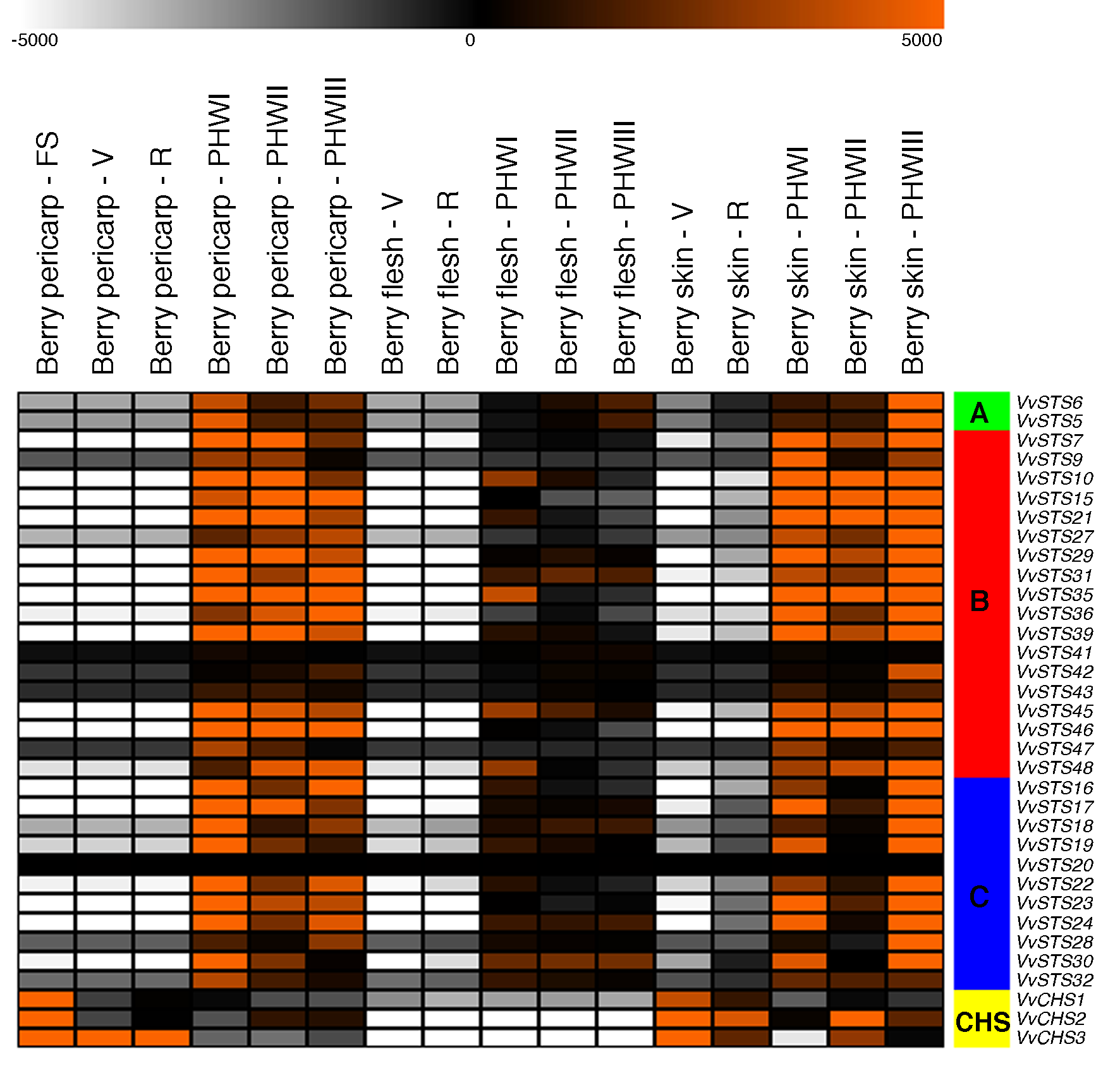

Supplement: Additional file 4 — Expression image of the completeVvSTSfamily in Corvina Berries undergoing withering process. This picture illustrate more in detail the expression of VvSTS and VvCHS genes in berry tissues (skin and flesh) during the last developmental phases and withering process. Expression values are normalised based on the mean expression value of each gene in all tissues/organs analysed. Different organs/tissues are displayed vertically above each column. VvSTS gene names are displayed to the right of each row and are clustered in different groups A, B, C according to protein homology as shown in Figure 2. [file 1471-2229-12-130-S4.tiff]
