# Supplementary material for: Genome-wide analysis of the grapevine stilbene synthase multigenic family: genomic organization and expression profiles upon biotic and abiotic stresses
Source: BMC Plant Biol. 2012 Aug 3;12:130. doi: 10.1186/1471-2229-12-130 (PMC3433347; doi:10.1186/1471-2229-12-130)
Supplement: Additional file 6 — Alignment of truncatedSTSprotein sequences. VvSTS1 and VvSTS4 deduced truncated proteins were aligned with a grapevine full-length STS (VvSTS48) and the three STS genes from P.densiflora (PdSTS1, PdSTS2 and PdSTS3). Alignment was obtained using MAFFT software and edited with GeneDoc software. The CHS/STS active site is highlighted in green. Stop codons are highlighted in red for those sequences considered of interest because they still contain the active site. [file 1471-2229-12-130-S6.pdf]

```
                   *        20         *        40         *        60         *        80
VvSTS48 : M--ASVEEFRNAQRAKGPATILAIGTATPDHCVYQSDYADYYFRVTKSEHMTELKKKFNRICDKSMIKKRYIHLTEEMLE :  78
VvSTS1  : M--ASVEDIRNAQRAKGPATILAIGTATPDNCVYQSDYADYYFRVTKSDHMTDLKKKFNRICEKSMIKKRYIHLTEEMLE :  78
VvSTS4  : M--ASVEDIRNAQHAKRLATILVIGTATPDNYAYQSDYADYYFRVTKSEHMTDLKKKFNRICEKSMIKKRYIHLTEEMLE :  78
PdSTS1  : MGGVDFEGFRKLQRADGFASILAIGTANPPNAVDQSTYPDYYFRITGNEHNTELKDKFKRICERSAIKQRYMYLTEEILK :  80
PdSTS2  : MGGVDFEGFRKLQRADGFASILAIGTANPPNAVDQSTYPDYYFRITGNEHNTELKDKFKRICERSAIKQRYMYLTEEILK :  80
PdSTS3  : MGGVDFEGFRKLQRADGFASILAIGTANPPNAVDQSTYPDYYFRITGNEHNTELKDKFKRICERSAIKQRYMYLTEEILK :  80
          M     E  R  QrA g A ILaIGTA P n v QS Y DYYFR T  eH T LK KF RICe S IK RY  LTEE L

                   *       100         *       120         *       140         *       160
VvSTS48 : EHPNIGAYM-APSLNIRQEIITAEVPRLGRDAALKALKEWGQPKSKITHLVFCTTSGVEMPGADYKLANLLGLETSVRRV : 157
VvSTS1  : EHPNMGAYM-APSLNIRQEIITAEVPKLGKEAALKALKEWGQPKSKITHLVFCTTSGVEMPGADYKLANLLGLETSVRRV : 157
VvSTS4  : EHPNMGAYM-APSLNIRQEIITTEVPKLGKEATLKALKEWGQPKSKITHLVFCTTSGVEMPGADYKLANLLGLETSVRRV : 157
PdSTS1  : KNPDVCAFVEVPSLDAGQAMLAMEVPRLAKEAAEKAIKEWGQSKSRITHLIFCSTTTPDLPGADFEVAKLLGLHPSVKRV : 160
PdSTS2  : KNPDVCAFVEVPSLDARQAMLAMEVPRLAKEAAEKAIHEWGQSKSGITHLIFCSTTTPDLPGADFEVAKLLGLHPSVKRV : 160
PdSTS3  : KNPDVCAFVEVPSLDARQAMLAMEVPRLAKEAAEKAIKEWGQSKSRITHLIFCSTTTPDLPGADFEVAKLLGLHPSVKRV : 160
            P   A    PSL  rQ     EVP L keAa KA kEWGQ KS ITHL FC T     PGAD   A LLGL  SV RV

                   *       180         *       200         *       220         *       240
VvSTS48 : MLYHQGCYAGGTVLRTAKDLAENNAGARVLVVCSEITVVTFRGPSEDALDSLVGQALFGDGSSAVIVGSDPDVSIERPLF : 237
VvSTS1  : MLYHQGCYAGGTVLRTAKDLAENNAGARVLVVCSEITVVTFRGPSETHLDSLVGQALFGDGSAAVIVGSDPDTSIE*~~~ : 233
VvSTS4  : MLYHQGCYAGGTVLRTAKDLAENNAGARVLVVCSEITVVTFRGPSETHLHSLVGQALFGDGSTTIIVGSDPDTSIERPLF : 237
PdSTS1  : GVFQHGCFAGGTVLRMAKDLAENNRGARVLVICSETTAVTFRGPSETHLDSLVGQALFGDGASALIVGADPIPQVEKACF : 240
PdSTS2  : GVFQHGCFAGGTVLRLAKDLAENNRGARVLVICSETTAVTFRGPSETHLDSLVGQALFGDGASALIVGADPIPQVEKACF : 240
PdSTS3  : GIFQHGCFAGGTVLRMAKDLAENNRGARVLVICSETTAVTFRGPSETHLDSLVGQALFGDGASALIVGADPIPQVEKACF : 240
               GC AGGTVLR AKDLAENN GARVLV CSE T VTFRGPSEthLdSLVGQALFGDG  a IVG DP    E   f

                   *       260         *       280         *       300         *       320
VvSTS48 : QLVSAAQTFIPNSAGAIAGNLREVGLTFHLWPNVPTLISENIEKCLTQAFDPLGISDWNSLFWIAHPGGPAILDAVEAKL : 317
VvSTS1  : ~~~~~~~~~~~~~~~~~~~~~~~~~~~~~~~~~~~~~~~~~~~~~~~~~~~~~~~~~~~~~~~~~~~~~~~~~~~~~~~~ :   -
VvSTS4  : QLISAAQTFIPNTQGAIVGNLREVGLTFHLWPNVPTLISENIEKCLTQAFAPLGISDWNSLFWIAHPGGPAILDAVEAKL : 317
PdSTS1  : EIVWTAQTVVPDSEGAIGGKVREVGLTFQLKGAVPDLISANIENCLVEAFSQFKISDWNKLFWVVHPGGRAILDRVEAKL : 320
PdSTS2  : EIVRTSQTVVPNSDGAIGGKVREVGLTFQLKGAVPDLISANIENCLVEAFSQFKICDWNKLFWVVHPGGRAILDRVEAKL : 320
PdSTS3  : EIVWTAQTVVPNSEGAIGGKVREVGLTFQLKGAVPDLISANIENCLVEAFTQFNIRLEQVVLGCSSRRTCHP*~~~~~~~ : 312
                qt  p   gai g  revgltf l   vp lis nie cl  af    i

                   *       340         *       360         *       380         *
VvSTS48 : NLEKKKLEATRHVLSEYGNMSSACVLFILDEMRKKSLKGENATTGEGLDWGVLFGFGPGLTIETVVLHSIPTVTN : 392
VvSTS1  : ~~~~~~~~~~~~~~~~~~~~~~~~~~~~~~~~~~~~~~~~~~~~~~~~~~~~~~~~~~~~~~~~~~~~~~~---- :   -
VvSTS4  : NLEKKKLEATRHIFSEYGNMSSACVLFILDEMRKKSLKEERTTTGKELD*~~~~~~~~~~~~~~~~~~~~~~~~~ : 366
PdSTS1  : NLDPTKLIPTRHVMSEYGNMSSACVHFILDQTRKASLQNGCSTSGEGLEMGVLFGFGPGLTIETVVLKSVPLQ*~ : 393
PdSTS2  : NLDPTKLIPTRHVMSEYGNMSSACVHFILDETRKASLRNGCSTTGEGLEMGVLFGFGPGLTIETVVLKSVPLQ*~ : 393
PdSTS3  : ~~~~~~~~~~~~~~~~~~~~~~~~~~~~~~~~~~~~~~~~~~~~~~~~~~~~~~~~~~~~~~~~~~~~~~~~~~~ :   -
```
